# Supplementary material for: HDAC6 as a Prognostic Factor and Druggable Target in HER2-Positive Breast Cancer
Source: Cancers (Basel). 2024 Nov 6;16(22):3752. doi: 10.3390/cancers16223752 (PMC11591923; doi:10.3390/cancers16223752)
Supplement: Supplementary file 1 [file cancers-16-03752-s001.zip › cancers-3285752-supplementary/TABLES/SupplementaryTableS1.pdf]

Table S1. PAM50 intrinsic subtype classification and Risk Of Recurrence (ROR) score and risk category for each patient.

Sample ID	Subtype	Nodal Status	Tumor Size	ROR Score	Risk Category	TIS score
C2	HER2-E	1	3	44	High	64
C3	Basal	1	0	56	High	82
C4	HER2-E	1	6	76	High	78
C5	LumB	1	0	76	High	55
C6	HER2-E	1	0	50	High	64
C7	LumA	1	2.3	49	High	77
C8	LumB	0	3.7	93	High	61
C9	LumB	1	0	65	High	82
C10	HER2-E		0	65		73
C11	LumB	1	4.8	79	High	51
C13	LumB	1	1.8	73	High	68
C14	LumB	1	2.1	89	High	7
C15	LumB	1	1.5	66	High	65
C16	HER2-E	1	5.5	72	High	65
C17	HER2-E	0	2.5	96	High	58
C19	HER2-E	1	4.8	82	High	83
C20	HER2-E		0	53		73
C21	LumB	1	7	66	High	70
C24	LumA	1	3	29	Intermediate	66
C25	Basal	0	2	48	Intermediate	83
C26	LumB	1	0	82	High	8
CTR1	LumA	0	3	34	Low	81
CTR2	LumA	0	1.5	43	Intermediate	78
CTR3	HER2-E	0	1.7	71	High	78
CTR4	HER2-E	1	0.8	50	High	64
CTR5	LumB	0	1.6	81	High	63
CTR6	HER2-E	0	0.7	73	High	79
CTR7	LumB	0	0.9	88	High	57
CTR8	LumB	0	1.1	82	High	91
CTR9	HER2-E	0	0	71	High	75
CTR10	HER2-E	0	1.5	70	High	58
CTR11	LumB	1	1.7	76	High	71
CTR12	LumA	1	1.1	22	Intermediate	67
CTR13	LumA	1	0	35	Intermediate	63
CTR14	HER2-E	1	2.7	87	High	74
CTR16	Basal	0	1.4	38	Low	77
CTR17	LumA	0	1.7	34	Low	64
CTR18	Basal	1	1.5	68	High	87
CTR19	LumA	0	2	25	Low	57
CTR20	LumB	0	1.5	87	High	79
CTR21	LumB	0	2.5	83	High	62

CTR22	LumB	1	1.6	72	High	67
CTR23	LumB	1	1.2	69	High	8
CTR24	LumA	1	0	11	Low	79
CTR25	HER2-E	1	1.8	73	High	78
CTR26	HER2-E	1	1.2	65	High	62
