# Supplementary material for: HDAC6 as a Prognostic Factor and Druggable Target in HER2-Positive Breast Cancer
Source: Cancers (Basel). 2024 Nov 6;16(22):3752. doi: 10.3390/cancers16223752 (PMC11591923; doi:10.3390/cancers16223752)
Supplement: Supplementary file 1 [file cancers-16-03752-s001.zip › cancers-3285752-supplementary/TABLES/SupplementaryTableS2.pdf]

Table S2. Summary of the differential gene expression analysis on gene signatures (unadjusted p-value <0.05). False discovery rate-adjusted p-values using the Benjamini-Yakutieli procedure are reported in the last column.

Signature name	Log2FC	95% CI	Unadj p-value	Adj p-value
B7-H3	0.46	0.14 - 0.78	0.005699	0.9753
PGR	-1.45	-2.76 - -0.15	0.02985	1
Hypoxia	0.31	0.02 - 0.60	0.03504	1
AR	-0.93	-1.80 - -0.07	0.03522	1
