# Supplementary material for: HDAC6 as a Prognostic Factor and Druggable Target in HER2-Positive Breast Cancer
Source: Cancers (Basel). 2024 Nov 6;16(22):3752. doi: 10.3390/cancers16223752 (PMC11591923; doi:10.3390/cancers16223752)
Supplement: Supplementary file 1 [file cancers-16-03752-s001.zip › cancers-3285752-supplementary/TABLES/supplementaryTableS3.pdf]

Table S3. Summary of the differential gene expression analysis (unadjusted p-value <0.05). False discovery rate-adjusted p-values using the Benjamini-Yakutieli procedure are reported in the last column.

Gene name	Log2FC	95% CI	Unadj p-value	Adj p-value
TMPRSS4	2.19	1.12 - 3.25	0.0001449	0.7594
GNG4	1.24	0.53 - 1.95	0.0009903	1
FOS	-1.39	-2.24 - -0.54	0.001989	1
PLCE1	0.85	0.28 - 1.41	0.003938	1
FGF9	1.04	0.34 - 1.74	0.00426	1
E2F5	0.52	0.16 - 0.88	0.005033	1
ELF3	0.90	0.28 - 1.52	0.005486	1
MSR1	0.51	0.15 - 0.87	0.006447	1
THBS1	0.59	0.17 - 1.02	0.006683	1
CD276	0.46	0.13 - 0.79	0.006919	1
HDAC6	0.33	0.09 - 0.56	0.007261	1
FAM124B	0.62	0.17 - 1.06	0.00736	1
ISM1	0.82	0.21 - 1.44	0.01005	1
HIF1A	0.47	0.12 - 0.83	0.01021	1
INHBA	0.48	0.10 - 0.85	0.01403	1
CLDN3	0.87	0.18 - 1.57	0.01496	1
PTGS2	-0.80	-1.45 - -0.15	0.01742	1
PAX8	0.75	0.14 - 1.37	0.01779	1
MAML2	0.42	0.07 - 0.76	0.01856	1
CDKN1A	0.46	0.08 - 0.85	0.01965	1
BNIP3	0.57	0.09 - 1.05	0.02021	1
GZMM	-0.59	-1.09 - -0.10	0.02021	1
FLRT3	0.98	0.16 - 1.81	0.02048	1
IL20RB	0.45	0.07 - 0.83	0.02245	1
SHMT2	0.50	0.07 - 0.92	0.02306	1
PRKACB	-0.4818	-0.89 - -0.07	0.02312	1
TNFSF10	-0.74	-1.38 - -0.10	0.02357	1
EYA2	0.77	0.11 - 1.44	0.02366	1
TLR4	0.43	0.06 - 0.79	0.02382	1
TLX1	0.71	0.10 - 1.33	0.02449	1
ANGPT1	0.41	0.05 - 0.78	0.02569	1
COL2A1	0.59	0.07 - 1.11	0.02683	1
CLDN7	0.51	0.06 - 0.95	0.02693	1
FLT3	-0.60	-1.14 - -0.07	0.028	1
PGR	-1.45	-2.74 - -0.16	0.02834	1
NOTCH2	0.28	0.03 - 0.54	0.03144	1
RPS6KA5	-0.35	-0.66 - -0.03	0.03161	1
CCL7	0.96	0.09 - 1.83	0.03204	1
AGTR1	-0.86	-1.64 - -0.08	0.03232	1
NR4A1	-0.97	-1.86 - -0.08	0.03313	1

BCL11A	-0.69	-1.32 - -0.05	0.03381	1
AR	-0.93	-1.79 - -0.07	0.03405	1
TSPAN7	-0.45	-0.86 - -0.03	0.03545	1
TFF3	-1.61	-3.10 - -0.11	0.03571	1
PLA2G2A	-0.95	-1.83 - -0.06	0.03594	1
FGF1	0.43	0.03 - 0.83	0.03639	1
VEGFA	0.50	0.03 - 0.97	0.03724	1
ATAD2	0.52	0.03 - 1.02	0.03745	1
S100A14	0.91	0.05 - 1.78	0.03772	1
WNT5B	0.50	0.03 - 0.98	0.03785	1
KLRK1	-0.55	-1.06 - -0.03	0.03787	1
BMP7	1.03	0.04 - 2.01	0.04083	1
CCL3L1	-0.57	-1.12 - -0.02	0.04125	1
LAD1	0.62	0.02 - 1.22	0.04184	1
PRLR	-0.65	-1.27 - -0.02	0.04243	1
GDF15	0.89	0.03 - 1.76	0.04321	1
CDCA7L	0.56	0.02 - 1.11	0.04335	1
TEK	0.37	0.01 - 0.73	0.04471	1
KIF23	0.51	0.01 - 1.01	0.04517	1
CXCL5	0.61	0.01 - 1.20	0.04545	1
FANCF	-0.32	-0.63 - -0.01	0.04649	1
JUN	-0.53	-1.06 - -0.003	0.04866	1
