# Supplementary material for: HDAC6 as a Prognostic Factor and Druggable Target in HER2-Positive Breast Cancer
Source: Cancers (Basel). 2024 Nov 6;16(22):3752. doi: 10.3390/cancers16223752 (PMC11591923; doi:10.3390/cancers16223752)
Supplement: Supplementary file 1 [file cancers-16-03752-s001.zip › cancers-3285752-supplementary/TABLES/SupplementaryTableS4.pdf]

Table S4. TCGA patients' characteristics (n=65)

Variable	n	%
**Age at diagnosis (years)**		
Mean ± sd	53.0 ± 11.7	
Min - max	27.0 – 84.0	
**Ethnicity**		
Hispanic or latino	2	3.7
Not hispanic or latino	52	96.3
missing	11	
**Race**		
American indian or Alaska native	1	1.6
Black or african american	17	27.9
White	43	70.5
missing	4	
**Cancer type**		
Breast invasive carcinoma (NOS)	1	1.5
Breast invasive ductal carcinoma	56	86.2
Breast invasive lobular carcinoma	8	12.3
**Tumor type**		
Infiltrating ductal carcinoma	56	86.2
Infiltrating lobular carcinoma	8	12.3
Other	1	1.5
**Subtype**		
BRCA-basal	5	8.3
BRCA-Her2	23	38.3
BRCA-Lum A	15	25.0
BRCA- Lum B	17	28.3
missing	5	
**M**		
CM0 (I+)	1	1.5
M0	53	81.5
MX	11	16.9
**N**		
N0	14	21.5
N0(I-)	12	18.5
N0(I+)	2	3.1
N1	8	12.3
N1A	7	10.8
N1B	2	3.1
N1M1	2	3.1
N2	3	4.6
N2A	8	12.3
N3	2	3.1
N3A	5	7.7

**T**		
T1	2	3.1
T1C	11	16.9
T2	42	64.6
T3	9	13.9
T4D	1	1.5
**Stage**		
I	5	7.7
IA	3	4.6
IIA	21	32.3
IIB	15	23.1
IIIA	14	21.5
IIIC	7	10.8
**Agent**		
Docetaxel	28	43.1
Paclitaxel	37	56.9
**Ki-67 (mRNA)**		
Median (IQ - IIIQ)	2146.3 (1743.3 – 3342.5)	
Min - max	200.5 – 8098.8	
**Ki-67 (prot)**		
Median (IQ - IIIQ)	-0.18 (-0.5 – 0.5)	
Min - max	-1.2 – 0.7	
missing	53	
**HADAC6**		
Median (IQ - IIIQ)	1064.9 (904.7 – 1213.2)	
Min - max	420.8 – 4100.3	

NOS: no special type; T, N, M are referred to the TNM staging system. T: size of primary tumor; N: lymph nodes involvement; M: distant metastasis.
