# Supplementary material for: HDAC6 as a Prognostic Factor and Druggable Target in HER2-Positive Breast Cancer
Source: Cancers (Basel). 2024 Nov 6;16(22):3752. doi: 10.3390/cancers16223752 (PMC11591923; doi:10.3390/cancers16223752)
Supplement: Supplementary file 1 [file cancers-16-03752-s001.zip › cancers-3285752-supplementary/TABLES/SupplementaryTableS5.pdf]

Table S5. Full list of NexturastatA target genes and of BC-associated genes

**NextA targets**	**BC-associated genes**										
ACE	ABCA1	CA2	CXCL8	FGF4	ID1	LINC00598	MIR20A	NOTCH1	PTENP1	SLX4	TPM1
ACHE	ABCA3	CA9	CXCR4	FGF6	IDH1	LINC00628	MIR21	NOTCH4	PTGS2	SMAD2	TPM2
ADAM10	ABCB1	CALM1	CYCS	FGF7	IDO1	LINC00636	MIR210	NOVA1	PTHLH	SMAD3	TPM3
ADAM17	ABCC1	CALML4	CYP17A1	FGF8	IFI16	LINC00901	MIR22	NPM1	PTK2	SMAD4	TRERF1
ADAM33	ABCC11	CASC2	CYP17A1-AS1	FGFR1	IFI27	LINC00958	MIR221	NQO1	PTK2B	SMAD7	TRIM24
ADAM8	ABCC12	CASC22	CYP19A1	FGFR2	IFNA2	LINC00993	MIR222	NQO2	PTK6	SMARCA4	TSG101
ADAMTS4	ABCC2	CASP2	CYP1A1	FGFR3	IFNB1	LINC01016	MIR27A	NR1H2	PTN	SMC4	TTC4
ADAMTS5	ABCG2	CASP3	CYP1A2	FGFR4	IFNG	LINC01089	MIR29B2	NR1I2	PTP4A1	SNAI1	TUBB
ADORA1	ABI1	CASP7	CYP1B1	FHIT	IGF1	LINC01116	MIR29C	NR3C1	PTPA	SNAI2	TUG1
ADORA2A	ABL1	CASP8	CYP24A1	FLNB	IGF1R	LINC01234	MIR30A	NR3C2	PTPN11	SNCG	TUNAR
ADORA2B	ABRAXAS1	CASP9	CYP2D6	FLT1	IGF2	LINC01405	MIR30E	NRG1	PTPN3	SNHG15	TWIST1
AKR1B10	ACTB	CAV1	CYP3A4	FLT4	IGF2R	LINC01671	MIR31	NRP1	PTPN9	SNHG16	TXN
ALDH2	ACTN4	CBFB	CYP3A5	FN1	IGFBP1	LINC02099	MIR31HG	NRP2	PTPRC	SNHG7	TYMP
ALK	ADAM11	CCAR2	CYTOR	FOS	IGFBP2	LINC02130	MIR320A	NT5E	PTPRG	SOCS3	TYMS
BMP1	ADAM12	CCAT1	DANCR	FOSL1	IGFBP3	LINC-ROR	MIR328	NTRK1	PTPRG-AS1	SOD2	UCA1
BRAF	ADAR	CCAT2	DDR1	FOXA1	IGFBP4	LINP1	MIR335	NTRK2	PTTG1	SOX2	UGDH
BRD4	ADARB2-AS1	CCDC170	DDR2	FOXC1	IGFBP5	LMTK3	MIR34A	NTRK3	PVT1	SOX2-OT	UHRF1
CA12	AFAP1	CCDC26	DDX3X	FOXC2-AS1	IGFBP6	LNCRNA-ATB	MIR373	NUS1	PXN	SOX4	UIMC1
CA2	AGR2	CCL2	DHFR	FOXCUT	IGKVa	LOX	MIR429	ODC1	PYHIN1	SP1	VDR
CA9	AGR3	CCL5	DIRC3	FOXM1	IKBKB	LOXL2	MIR451A	OGG1	QSOX1	SPARC	VEGFA
CAMK2D	AHR	CCN1	DKC1	FOXO1	IKBKE	LRATD2	MIR497	OLA1	RAB25	SPDEF	VEGFC
CAPN1	AKAP13	CCN2	DKK1	FOXO3	IL10	LRRC56	MIR499A	OR3A4P	RAC1	SPHK1	VEGFD

CDC25A	AKIP1	CCN4	DLEU1	FOXP3	IL11	LSM1	MIR502	P3H2	RAC3	SPHK2	VIM
CDC25B	AKR1C3	CCNA2	DNMT1	FSCN1	IL17A	LSP1	MIR510	PAK1	RAD21	SPP1	VTN
CDK2	AKT1	CCNB1	DNMT3B	FUS	IL1B	LYVE1	MIR520C	PALB2	RAD50	SPRY4-IT1	VWA5A
CDK4	AKT2	CCNB2	DPP10-AS1	FZR1	IL2	MACF1	MIR661	PANDAR	RAD51	SRA1	WASF3
CHEK1	ALCAM	CCND1	DPYD	GAB1	IL2RA	MACROD1	MIR663A	PARP1	RAD51B	SRC	WNT1
CMA1	ALDH1A1	CCND2	DRAIC	GADD45A	IL3	MACROH2A1	MIR9-1	PAXIP1	RAD51C	SRRM3	WNT10B
CYP19A1	ALK	CCND3	DSCAM-AS1	GAPDH	IL4	MAD1L1	MIR9-3	PBOV1	RAD51D	SSTR2	WNT3
CYP24A1	AMPH	CCNE1	DUSP1	GAS5	IL6	MAD2L1	MIR96	PCID2	RAD51L3-RFFL	ST14	WNT5A
DRD2	ANAPC5	CCNE2	DUSP3	GATA3	ING1	MAGED2	MIR98	PCNA	RAD52	ST18	WT1
DRD3	ANGPT2	CCNG1	E2F1	GDF15	INPPL1	MAGI2-AS3	MIRLET7A1	PDCD1	RAD54L	ST8SIA1	WT1-AS
DRD4	ANKRD30A	CCNH	E2F3	GH1	INS	MALAT1	MIRLET7A3	PDCD4	RAF1	ST8SIA6-AS1	WWC1
DUSP3	ANXA1	CCNT2	ECM1	GHRH	INSR	MANCR	MIRLET7D	PDCD4-AS1	RANBP9	STARD13	WWOX
EPHB4	ANXA2	CCR6	ECT2	GJA1	IRAIN	MAP2K1	MIRLET7F1	PDGFA	RARA	STAT1	WWTR1
EPHX2	ANXA5	CCR7	EDNRA	GLI1	IRS1	MAP2K3	MIRLET7F2	PDGFB	RARB	STAT3	XBP1
ERN1	AOPEP	CD24	EFNA1	GMNN	IRS2	MAP2K4	MIRLET7I	PDGFRA	RARG	STAT5A	XDH
F3	APC	CD274	EFNA3	GNAS	ITGA3	MAP2K5	MKI67	PDGFRB	RARS1	STAT5B	XIAP
FLT1	APEX1	CD44	EGF	GNG3	ITGA6	MAP3K1	MLH1	PDLIM2	RASSF1	STIM1	XIST
FNTA FNTB	APOD	CDC25A	EGFR	GNRH1	ITGAV	MAPK1	MMP1	PDPN	RASSF1-AS1	STK11	XPO1
GCGR	APPBP2	CDC25C	EGOT	GPC6	ITGB1	MAPK10	MMP11	PEA15	RB1	STMN1	XRCC1
GIPR	AR	CDC27	EGR1	GPER1	ITGB3	MAPK12	MMP13	PELP1	RB1CC1	STRADA	XRCC2
GRM2	AREG	CDC42	EHMT1	GPNMB	ITGB3BP	MAPK14	MMP14	PER1	RBBP4	STS	XRCC3
HCK	ARFGEF3	CDC5L	EI24	GPR161	ITGB4	MAPK3	MMP17	PFN1	RBBP8	STXBP5-AS1	XRCC5
HDAC1	ARHGEF2	CDC73	EIF3A	GPX1	JADRR	MAPK7	MMP2	PGR	RBX1	SULF2	XRCC6
HDAC10	ARID4B	CDCA7	EIF3E	GRB2	JAG1	MAPK8	MMP3	PHB1	RELA	SULT1A1	
HDAC11	ARRDC3	CDH1	EIF3H	GRB7	JAK2	MAPK9	MMP7	PHLDA2	RELB	SULT1E1	YBX1

HDAC2	ATAD2	CDH13	EIF4E	GREB1	JUN	MATK	MMP9	PIK3CA	RERG	SUSD2	ZEB1
HDAC3	ATF2	CDH2	EIF4EBP1	GRM1	KCNK15-AS1	MCL1	MNX1-AS1	PIK3CB	REST	SVEP1	ZEB2
HDAC4	ATM	CDH3	ELAVL1	GRN	KCNQ1OT1	MCM2	MRC2	PIK3CG	RET	SYK	ZFAS1
HDAC6	ATR	CDK1	ELF3	GRP	KDM3B	MDM2	MRE11	PIK3R1	RHNO1	SYP	ZFHX3
HDAC7	AURKA	CDK2	ELOC	GSK3B	KDM4B	MED1	MSH2	PIK3R4	RHOA	TACC1	ZFP36
HDAC8	AURKB	CDK4	EMSY	GSN	KDM4C	MED12	MSH3	PIN1	RHOB	TACC2	ZNF350
HDAC9	AXIN2	CDK6	ENAH	GSTM1	KDM5B	MED14	MSH6	PINK1-AS	RHOBTB2	TCF7L2	ZNF365
HSD17B3	AXL	CDK7	ENG	GSTP1	KDR	MEG3	MSR1	PIP	RHOC	TCIM	ZNF667-AS1
HSP90AA1	BAG1	CDKN1A	ENO1	H19	KEAP1	MET	MST1R	PKD1P6-NPIPP1	RLIM	TCP1	ZNF703
HSP90AB1	BAK1	CDKN1B	EP300	H2AC18	KIAA0100	MIAT	MTA1	PLA2G10	RMST	TDGF1	
HTT	BANP	CDKN2A	EPAS1	H2AX	KIAA1671	MIR101-1	MTA3	PLAU	RNF11	TEK	
IMPDH1	BAP1	CDKN2B-AS1	EPB41L4A-DT	HBEGF	KIF15	MIR10B	MT-CYB	PLAUR	RNF146	TERT	
JAK2	BARD1	CDKN2C	EPCAM	HBP1	KISS1	MIR122	MTDH	PLCB1	ROBO1	TFAP2A	
KCNA5	BAX	CDKN3	EPHA2	HDAC1	KIT	MIR124-3	MTHFR	PLCG1	ROCK1	TFAP2C	
MAPKAPK2	BCAR1	CEACAM5	EPHA3	HDAC2	KITLG	MIR125A	MTOR	PLD2	ROCK2	TFF1	
MDM2	BCAR3	CEBPB	EPHA4	HDAC4	KLC1	MIR125B1	MT-TE	PLEKHA8	ROR1	TGFA	
MET	BCAR4	CEMIP	EPHB4	HDAC5	KLF17	MIR126	MT-TT	PLK1	ROR2	TGFB1	
MME	BCAS1	CEP85L	EPOR	HDAC6	KLF3-AS1	MIR127	MTUS1	PMS2	RPS6KA3	TGFB2	
MMP10	BCAS2	CERNA2	EPSTI1	HDAC9	KLF4	MIR128-1	MUC1	POLE	RPS6KB1	TGFB3	
MMP12	BCAS3	CHEK1	ERBB2	HEATR6	KLF5	MIR128-2	MUCL1	POLQ	RRAS	TGFBR1	
MMP14	BCAS4	CHEK2	ERBB3	HERC2	KLK10	MIR136	MUTYH	POSTN	RRAS2	TGFBR2	
MMP3	BCDIN3D	CIB1	ERBB4	HFE	KLK13	MIR141	MVP	POU5F1	RUNX2	TGFBR3	
MMP7	BCL2	CKS1B	ERCC1	HGF	KLK14	MIR143	MYB	PPARA	RUNX3	TGIF1	
NCOR1	BCL2L1	CLCA2	ERCC2	HIF1A	KLK3	MIR145	MYC	PPARG	RYBP	THBS1	

ODC1	BCL2L11	CLDN4	ERCC3	HIF1A-AS2	KLK9	MIR146A	MYH9	PPM1D	S100A14	THBS4
PDE3B	BCYRN1	CLDN7	ERCC6	HLA-G	KLLN	MIR146B	MYLK	PPP1R14C	S100A2	TIE1
PDE4A	BDNF	CLDND1	EREG	HMGA1	KRAS	MIR148A	NAT1	PPP1R1B	S100A4	TIMELESS
PDE4B	BECN1	CLOCK	ERVH48-1	HMGB1	KRT14	MIR149	NAT2	PPP1R3A	S100A6	TIMP1
PDE4C	BEX2	CLU	ESR1	HMMR	KRT18	MIR152	NBAT1	PPP2R1A	S100A7	TIMP2
PDGFRB	BIN1	COL11A1	ESR2	HMMR-AS1	KRT19	MIR155	NBN	PPP2R2A	SAFB	TIMP3
PI4KB	BIRC5	COMT	ESRRA	HMOX1	KRT20	MIR17	NCOA1	PPP5C	SAFB2	TIMP4
PIM2	BMI1	COX7A2L	ESRRG	HNF1A	KRT5	MIR181A1	NCOA2	PRDM14	SCGB1D2	TJP1
PIM3	BMP1	CREB1	ETS1	HNRNPK	KRT7	MIR181A2	NCOA3	PRDM2	SCGB2A2	TMPRSS6
PLK1	BMP10	CREBBP	ETV1	HNRNPUL2-BSCL2	KRT8	MIR181B1	NCOA6	PRKAA1	SCGB3A1	TNC
PRF1	BMP2	CRNDE	ETV4	HOTAIR	KSR1	MIR181D	NCOR1	PRKAA2	SDC1	TNF
PRKDC	BMP4	CSF1	EZH2	HOTTIP	LASP1	MIR182	NCOR2	PRKACA	SEMA3A	TNFRSF10A
PSEN2 PSENEN NCSTN APH1A PSEN1 APH1B	BMP6	CSF1R	EZR	HOXA11-AS	LCN2	MIR18A	NEAT1	PRKCA	SERPINA3	TNFRSF10B
PTGS2	BMP7	CSF2	F2R	HOXA5	LDB1	MIR191	NEK2	PRKCB	SERPINB2	TNFRSF11A
RAC1	BMPR1A	CSF3	FABP3	HOXA-AS2	LDHA	MIR193B	NET1	PRKCD	SERPINB5	TNFRSF11B
RET	BPHL	CSK	FABP4	HPR	LEP	MIR195	NF1	PRKCQ	SERPINE1	TNFSF10
ROCK2	BPIFA4P	CSN2	FAM3C	HPSE	LEPR	MIR196A1	NF2	PRKD1	SF3B1	TNFSF11
S1PR1	BRAF	CST6	FANCC	HRAS	LGALS1	MIR196A2	NFE2L2	PRKD3	SF3B2	TOP1
S1PR3	BRCA1	CTCF	FANCD2	HSD17B1	LGALS3	MIR199A1	NFKB1	PRL	SFN	TOP2A
SF3B3	BRCA2	CTLA4	FANCM	HSP90AA1	LHCGR	MIR199B	NFKBIA	PRLR	SGK3	TOX3
SIGMAR1	BRIP1	CTNNB1	FAS	HSPA4	LIFR	MIR200A	NGF	PRMT2	SGO1	TP53
SPHK1	BRMS1	CTNND1	FASLG	HSPA5	LIMK1	MIR200B	NGFR	PRMT7	SHBG	TP53BP1
SPHK2	BRMS1L	CTSB	FASN	HSPA8	LINC00052	MIR200C	NKILA	PRRC2A	SHC1	TP53COR1

SRC	BSCL2	CTSD	FBLN1	HSPB1	LINC00160	MIR202	NKX2-1	PRSS50	SIRT1	TP63
SYK	BSG	CTTN	FBXW7	HSPB2	LINC00339	MIR203A	NME1	PSEN2	SKP2	TP73
TBXAS1	BTC	CUEDC2	FGF1	HSPB8	LINC00472	MIR204	NNT-AS1	PSIP1	SLC22A18	TP73-AS1
TSPO	BTK	CUL1	FGF14-AS2	IBSP	LINC00511	MIR205	NOP16	PSMA1	SLC2A1	TPD52
VCP	BUB1B	CUL3	FGF2	ICAM1	LINC00520	MIR2052HG	NORAD	PSMB5	SLC39A6	TPD52L1
WEE1	C11orf65	CXCL12	FGF3	ICAM5	LINC00574	MIR206	NOS2	PTEN	SLC9A3R1	TPD52L2
