# Supplementary material for: HDAC6 as a Prognostic Factor and Druggable Target in HER2-Positive Breast Cancer
Source: Cancers (Basel). 2024 Nov 6;16(22):3752. doi: 10.3390/cancers16223752 (PMC11591923; doi:10.3390/cancers16223752)
Supplement: Supplementary file 1 [file cancers-16-03752-s001.zip › cancers-3285752-supplementary/TABLES/SupplementaryTableS6.pdf]

GO biological process complete	Homo sapiens - REFLIST (20592)	upload_1 (40)	upload_1 (expected)	upload_1 (over/under)	upload_1 (fold Enrichment)	upload_1 (raw P-value)	upload_1 (FDR)	Colona 1
positive regulation of phosphorylation (GO:0042327)	632	19	1.23	+	15.48	1.66E-18	2.54E-14	
regulation of phosphate metabolic process (GO:0019220)	1133	22	2.20	+	10.00	1.02E-17	7.80E-14	
positive regulation of phosphorus metabolic process (GO:0010562)	699	19	1.36	+	13.99	1.02E-17	5.23E-14	
positive regulation of phosphate metabolic process (GO:0045937)	699	19	1.36	+	13.99	1.02E-17	3.92E-14	
regulation of phosphorus metabolic process (GO:0051174)	1134	22	2.20	+	9.99	1.03E-17	3.18E-14	
regulation of phosphorylation (GO:0042325)	1003	21	1.95	+	10.78	1.78E-17	4.57E-14	
regulation of cell migration (GO:0030334)	936	19	1.82	+	10.45	1.93E-15	4.23E-12	
positive regulation of cellular process (GO:0048522)	5698	35	11.07	+	3.16	4.37E-15	8.38E-12	
positive regulation of biological process (GO:0048518)	6211	36	12.06	+	2.98	4.45E-15	7.58E-12	
regulation of cell motility (GO:2000145)	996	19	1.93	+	9.82	5.84E-15	8.97E-12	
positive regulation of protein phosphorylation (GO:0001934)	585	16	1.14	+	14.08	7.26E-15	1.01E-11	
regulation of locomotion (GO:0040012)	1038	19	2.02	+	9.42	1.22E-14	1.56E-11	
phosphorylation (GO:0016310)	744	17	1.45	+	11.76	1.46E-14	1.72E-11	
positive regulation of cell population proliferation (GO:0008284)	1255	20	2.44	+	8.20	2.40E-14	2.63E-11	
regulation of protein phosphorylation (GO:0001932)	926	18	1.80	+	10.01	2.88E-14	2.95E-11	
positive regulation of protein modification process (GO:0031401)	793	17	1.54	+	11.04	4.03E-14	3.87E-11	

**protein metabolic process (GO:0019538)**	3620	29	7.03	+	4.12	4.14E-14	3.74 E-11
**positive regulation of cell migration (GO:0030335)**	541	15	1.05	+	14.27	5.15E-14	4.39 E-11
**metabolic process (GO:0008152)**	8001	38	15.54	+	2.44	7.96E-14	6.43 E-11
**positive regulation of cell motility (GO:2000147)**	568	15	1.10	+	13.60	1.03E-13	7.87 E-11
**positive regulation of locomotion (GO:0040017)**	583	15	1.13	+	13.25	1.48E-13	1.08 E-10
**response to stimulus (GO:0050896)**	8194	38	15.92	+	2.39	1.91E-13	1.33 E-10
**protein phosphorylation (GO:0006468)**	493	14	.96	+	14.62	3.17E-13	2.11 E-10
**organonitrogen compound metabolic process (GO:1901564)**	4684	31	9.10	+	3.41	3.63E-13	2.32 E-10
**regulation of protein modification process (GO:0031399)**	1272	19	2.47	+	7.69	4.42E-13	2.71 E-10
**positive regulation of protein metabolic process (GO:0051247)**	1285	19	2.50	+	7.61	5.28E-13	3.12 E-10
**regulation of protein metabolic process (GO:0051246)**	2230	23	4.33	+	5.31	9.47E-13	5.39 E-10
**response to chemical (GO:0042221)**	3782	28	7.35	+	3.81	1.43E-12	7.84 E-10
**response to organic substance (GO:0010033)**	2585	24	5.02	+	4.78	2.06E-12	1.09 E-09
**peptidyl-amino acid modification (GO:0018193)**	574	14	1.11	+	12.56	2.35E-12	1.20 E-09
**cellular response to chemical stimulus (GO:0070887)**	2391	23	4.64	+	4.95	4.05E-12	2.01 E-09
**protein modification process (GO:0036211)**	2137	22	4.15	+	5.30	4.28E-12	2.05 E-09
**response to oxygen-containing compound (GO:1901700)**	1670	20	3.24	+	6.17	4.62E-12	2.15 E-09
**positive regulation of cellular metabolic process (GO:0031325)**	3349	26	6.51	+	4.00	7.15E-12	3.23 E-09
**cellular response to oxygen-containing compound (GO:1901701)**	1138	17	2.21	+	7.69	1.21E-11	5.30 E-09
**response to lipid (GO:0033993)**	850	15	1.65	+	9.08	2.91E-11	1.24 E-08

**macromolecule modification (GO:0043412)**	2358	22	4.58	+	4.80	3.00E-11	1.25 E-08
**regulation of nitrogen compound metabolic process (GO:0051171)**	5483	31	10.65	+	2.91	3.03E-11	1.22 E-08
**primary metabolic process (GO:0044238)**	6966	34	13.53	+	2.51	3.66E-11	1.44 E-08
**response to organic cyclic compound (GO:0014070)**	869	15	1.69	+	8.89	3.95E-11	1.52 E-08
**regulation of multicellular organismal process (GO:0051239)**	2965	24	5.76	+	4.17	3.95E-11	1.48 E-08
**multicellular organism development (GO:0007275)**	3953	27	7.68	+	3.52	3.99E-11	1.46 E-08
**cellular response to stimulus (GO:0051716)**	6476	33	12.58	+	2.62	4.05E-11	1.45 E-08
**regulation of response to stimulus (GO:0048583)**	3976	27	7.72	+	3.50	4.58E-11	1.60 E-08
**response to endogenous stimulus (GO:0009719)**	1447	18	2.81	+	6.40	4.78E-11	1.63 E-08
**positive regulation of molecular function (GO:0044093)**	1248	17	2.42	+	7.01	5.07E-11	1.69 E-08
**positive regulation of nitrogen compound metabolic process (GO:0051173)**	3015	24	5.86	+	4.10	5.64E-11	1.84 E-08
**regulation of cellular metabolic process (GO:0031323)**	6066	32	11.78	+	2.72	5.69E-11	1.82 E-08
**phosphate-containing compound metabolic process (GO:0006796)**	1686	19	3.28	+	5.80	5.86E-11	1.84 E-08
**regulation of metabolic process (GO:0019222)**	6584	33	12.79	+	2.58	6.63E-11	2.03 E-08
**regulation of primary metabolic process (GO:0080090)**	5649	31	10.97	+	2.83	6.92E-11	2.08 E-08
**regulation of cell population proliferation (GO:0042127)**	2198	21	4.27	+	4.92	7.26E-11	2.14 E-08
**phosphorus metabolic process (GO:0006793)**	1712	19	3.33	+	5.71	7.62E-11	2.21 E-08
**positive regulation of metabolic process (GO:0009893)**	3736	26	7.26	+	3.58	8.98E-11	2.55 E-08
**regulation of molecular function (GO:0065009)**	2050	20	3.98	+	5.02	1.86E-10	5.19 E-08

**regulation of cellular component biogenesis (GO:0044087)**	973	15	1.89	+	7.94	1.88E-10	5.14 E-08
**system development (GO:0048731)**	3538	25	6.87	+	3.64	2.15E-10	5.80 E-08
**regulation of kinase activity (GO:0043549)**	537	12	1.04	+	11.50	3.17E-10	8.39 E-08
**organic substance metabolic process (GO:0071704)**	7478	34	14.53	+	2.34	3.26E-10	8.48 E-08
**positive regulation of multicellular organismal process (GO:0051240)**	1647	18	3.20	+	5.63	3.90E-10	9.98 E-08
**transmembrane receptor protein tyrosine kinase signaling pathway (GO:0007169)**	420	11	.82	+	13.48	3.90E-10	9.82 E-08
**regulation of catalytic activity (GO:0050790)**	1433	17	2.78	+	6.11	4.26E-10	1.05 E-07
**regulation of macromolecule metabolic process (GO:0060255)**	6058	31	11.77	+	2.63	4.73E-10	1.15 E-07
**regulation of cellular component organization (GO:0051128)**	2436	21	4.73	+	4.44	4.93E-10	1.18 E-07
**regulation of response to stress (GO:0080134)**	1452	17	2.82	+	6.03	5.21E-10	1.23 E-07
**anatomical structure development (GO:0048856)**	5214	29	10.13	+	2.86	5.59E-10	1.30 E-07
**response to nitrogen compound (GO:1901698)**	1060	15	2.06	+	7.28	6.05E-10	1.39 E-07
**developmental process (GO:0032502)**	5723	30	11.12	+	2.70	8.09E-10	1.83 E-07
**regulation of response to external stimulus (GO:0032101)**	1086	15	2.11	+	7.11	8.42E-10	1.87 E-07
**regulation of signaling (GO:0023051)**	3428	24	6.66	+	3.60	8.47E-10	1.86 E-07
**positive regulation of macromolecule metabolic process (GO:0010604)**	3431	24	6.66	+	3.60	8.63E-10	1.87 E-07
**regulation of cell communication (GO:0010646)**	3433	24	6.67	+	3.60	8.73E-10	1.86 E-07
**positive regulation of cellular component organization (GO:0051130)**	1118	15	2.17	+	6.91	1.25E-09	2.63 E-07
**regulation of intracellular signal transduction (GO:1902531)**	1773	18	3.44	+	5.23	1.27E-09	2.64 E-07

**animal organ development (GO:0048513)**	2859	22	5.55	+	3.96	1.27E-09	2.60 E-07
**regulation of cellular process (GO:0050794)**	11189	39	21.73	+	1.79	1.29E-09	2.61 E-07
**regulation of localization (GO:0032879)**	2039	19	3.96	+	4.80	1.47E-09	2.93 E-07
**cellular metabolic process (GO:0044237)**	6383	31	12.40	+	2.50	1.96E-09	3.86 E-07
**nitrogen compound metabolic process (GO:0006807)**	6402	31	12.44	+	2.49	2.13E-09	4.14 E-07
**regulation of MAPK cascade (GO:0043408)**	649	12	1.26	+	9.52	2.60E-09	4.99 E-07
**response to organonitrogen compound (GO:0010243)**	988	14	1.92	+	7.29	2.64E-09	5.00 E-07
**regulation of transferase activity (GO:0051338)**	652	12	1.27	+	9.47	2.74E-09	5.12 E-07
**cellular response to endogenous stimulus (GO:0071495)**	1187	15	2.31	+	6.51	2.81E-09	5.19 E-07
**protein deacetylation (GO:0006476)**	25	5	.05	+	> 100	2.91E-09	5.31 E-07
**positive regulation of transferase activity (GO:0051347)**	384	10	.75	+	13.41	2.93E-09	5.29 E-07
**regulation of signal transduction (GO:0009966)**	3018	22	5.86	+	3.75	3.57E-09	6.38 E-07
**macromolecule metabolic process (GO:0043170)**	5672	29	11.02	+	2.63	4.65E-09	8.21 E-07
**peptidyl-tyrosine phosphorylation (GO:0018108)**	65	6	.13	+	47.52	4.68E-09	8.17 E-07
**peptidyl-tyrosine modification (GO:0018212)**	67	6	.13	+	46.10	5.55E-09	9.58 E-07
**regulation of biological process (GO:0050789)**	11738	39	22.80	+	1.71	6.12E-09	1.04 E-06
**response to stress (GO:0006950)**	3436	23	6.67	+	3.45	6.33E-09	1.07 E-06
**multicellular organismal process (GO:0032501)**	6730	31	13.07	+	2.37	8.17E-09	1.36 E-06
**anatomical structure morphogenesis (GO:0009653)**	2261	19	4.39	+	4.33	8.18E-09	1.35 E-06
**positive regulation of kinase activity (GO:0033674)**	311	9	.60	+	14.90	8.32E-09	1.36 E-06
**regulation of programmed cell death (GO:0043067)**	1522	16	2.96	+	5.41	9.48E-09	1.53 E-06
**positive regulation of catalytic activity (GO:0043085)**	905	13	1.76	+	7.39	9.69E-09	1.55 E-06

**cellular response to organic substance (GO:0071310)**	1764	17	3.43	+	4.96	9.83E-09	1.56 E-06
**response to external stimulus (GO:0009605)**	2290	19	4.45	+	4.27	1.01E-08	1.58 E-06
**protein deacylation (GO:0035601)**	34	5	.07	+	75.71	1.16E-08	1.80 E-06
**regulation of macromolecule biosynthetic process (GO:0010556)**	5036	27	9.78	+	2.76	1.18E-08	1.81 E-06
**response to abiotic stimulus (GO:0009628)**	1114	14	2.16	+	6.47	1.20E-08	1.82 E-06
**response to peptide (GO:1901652)**	459	10	.89	+	11.22	1.54E-08	2.32 E-06
**regulation of proteolysis (GO:0030162)**	605	11	1.18	+	9.36	1.62E-08	2.41 E-06
**macromolecule deacylation (GO:0098732)**	37	5	.07	+	69.57	1.71E-08	2.52 E-06
**positive regulation of epithelial cell migration (GO:0010634)**	151	7	.29	+	23.86	2.04E-08	2.98 E-06
**positive regulation of proteolysis (GO:0045862)**	347	9	.67	+	13.35	2.09E-08	3.03 E-06
**regulation of cellular biosynthetic process (GO:0031326)**	5167	27	10.04	+	2.69	2.12E-08	3.04 E-06
**cellular response to nitrogen compound (GO:1901699)**	625	11	1.21	+	9.06	2.25E-08	3.19 E-06
**enzyme-linked receptor protein signaling pathway (GO:0007167)**	626	11	1.22	+	9.05	2.28E-08	3.21 E-06
**regulation of biosynthetic process (GO:0009889)**	5198	27	10.10	+	2.67	2.43E-08	3.39 E-06
**regulation of nucleobase-containing compound metabolic process (GO:0019219)**	4055	24	7.88	+	3.05	2.68E-08	3.71 E-06
**biological regulation (GO:0065007)**	12153	39	23.61	+	1.65	3.17E-08	4.35 E-06
**regulation of mitotic cell cycle (GO:0007346)**	497	10	.97	+	10.36	3.21E-08	4.37 E-06
**regulation of developmental process (GO:0050793)**	2466	19	4.79	+	3.97	3.39E-08	4.56 E-06
**negative regulation of biological process (GO:0048519)**	5284	27	10.26	+	2.63	3.52E-08	4.70 E-06
**response to growth factor (GO:0070848)**	505	10	.98	+	10.19	3.72E-08	4.93 E-06

**response to oxidative stress (GO:0006979)**	372	9	.72	+	12.45	3.75E-08	4.92 E-06
**regulation of gene expression (GO:0010468)**	4909	26	9.54	+	2.73	4.09E-08	5.32 E-06
**positive regulation of protein localization to nucleus (GO:1900182)**	96	6	.19	+	32.17	4.21E-08	5.44 E-06
**cellular response to lipid (GO:0071396)**	527	10	1.02	+	9.77	5.51E-08	7.05 E-06
**positive regulation of cell communication (GO:0010647)**	1748	16	3.40	+	4.71	6.59E-08	8.36 E-06
**positive regulation of signaling (GO:0023056)**	1750	16	3.40	+	4.71	6.69E-08	8.42 E-06
**intracellular signal transduction (GO:0035556)**	1525	15	2.96	+	5.06	7.83E-08	9.78 E-06
**positive regulation of macromolecule biosynthetic process (GO:0010557)**	2608	19	5.07	+	3.75	8.38E-08	1.04 E-05
**positive regulation of transport (GO:0051050)**	903	12	1.75	+	6.84	9.54E-08	1.17 E-05
**histone deacetylation (GO:0016575)**	19	4	.04	+	> 100	1.04E-07	1.27 E-05
**signaling (GO:0023052)**	5133	26	9.97	+	2.61	1.07E-07	1.30 E-05
**positive regulation of developmental process (GO:0051094)**	1332	14	2.59	+	5.41	1.10E-07	1.32 E-05
**response to xenobiotic stimulus (GO:0009410)**	431	9	.84	+	10.75	1.28E-07	1.52 E-05
**regulation of nitric oxide biosynthetic process (GO:0045428)**	58	5	.11	+	44.38	1.37E-07	1.62 E-05
**positive regulation of cellular biosynthetic process (GO:0031328)**	2690	19	5.23	+	3.64	1.38E-07	1.61 E-05
**positive regulation of biosynthetic process (GO:0009891)**	2710	19	5.26	+	3.61	1.55E-07	1.80 E-05
**regulation of nitric oxide metabolic process (GO:0080164)**	60	5	.12	+	42.90	1.60E-07	1.85 E-05
**signal transduction (GO:0007165)**	4829	25	9.38	+	2.67	1.64E-07	1.88 E-05
**cell communication (GO:0007154)**	5285	26	10.27	+	2.53	2.01E-07	2.29 E-05
**regulation of focal adhesion assembly (GO:0051893)**	63	5	.12	+	40.86	2.01E-07	2.27 E-05

**regulation of cell-substrate junction assembly (GO:0090109)**	63	5	.12	+	40.86	2.01E-07	2.26 E-05
**response to ketone (GO:1901654)**	214	7	.42	+	16.84	2.03E-07	2.26 E-05
**response to hormone (GO:0009725)**	780	11	1.52	+	7.26	2.05E-07	2.26 E-05
**positive regulation of cell cycle (GO:0045787)**	326	8	.63	+	12.63	2.12E-07	2.33 E-05
**regulation of cytokine production (GO:0001817)**	784	11	1.52	+	7.22	2.16E-07	2.35 E-05
**positive regulation of gene expression (GO:0010628)**	1183	13	2.30	+	5.66	2.16E-07	2.34 E-05
**positive regulation of nucleocytoplasmic transport (GO:0046824)**	64	5	.12	+	40.22	2.17E-07	2.33 E-05
**regulation of cell-substrate adhesion (GO:0010810)**	217	7	.42	+	16.61	2.23E-07	2.37 E-05
**positive regulation of MAPK cascade (GO:0043410)**	464	9	.90	+	9.99	2.36E-07	2.50 E-05
**negative regulation of cellular process (GO:0048523)**	4926	25	9.57	+	2.61	2.47E-07	2.60 E-05
**cellular response to hydrogen peroxide (GO:0070301)**	66	5	.13	+	39.00	2.51E-07	2.62 E-05
**regulation of transport (GO:0051049)**	1668	15	3.24	+	4.63	2.51E-07	2.60 E-05
**cellular response to reactive oxygen species (GO:0034614)**	133	6	.26	+	23.22	2.67E-07	2.75 E-05
**regulation of cell-substrate junction organization (GO:0150116)**	68	5	.13	+	37.85	2.88E-07	2.95 E-05
**positive regulation of response to stimulus (GO:0048584)**	2229	17	4.33	+	3.93	3.02E-07	3.07 E-05
**regulation of epithelial cell migration (GO:0010632)**	229	7	.44	+	15.74	3.17E-07	3.20 E-05
**cellular response to organic cyclic compound (GO:0071407)**	490	9	.95	+	9.46	3.70E-07	3.71 E-05
**regulation of apoptotic process (GO:0042981)**	1477	14	2.87	+	4.88	3.87E-07	3.86 E-05
**positive regulation of cytokine production (GO:0001819)**	494	9	.96	+	9.38	3.95E-07	3.92 E-05
**regulation of cellular response to stress (GO:0080135)**	657	10	1.28	+	7.84	4.11E-07	4.05 E-05

**positive regulation of intracellular signal transduction (GO:1902533)**	1037	12	2.01	+	5.96	4.17E-07	4.08 E-05
**regulation of reactive oxygen species metabolic process (GO:2000377)**	144	6	.28	+	21.45	4.18E-07	4.06 E-05
**positive regulation of cellular component biogenesis (GO:0044089)**	509	9	.99	+	9.10	5.06E-07	4.88 E-05
**regulation of protein localization to nucleus (GO:1900180)**	149	6	.29	+	20.73	5.07E-07	4.87 E-05
**response to peptide hormone (GO:0043434)**	377	8	.73	+	10.92	6.24E-07	5.95 E-05
**regulation of inflammatory response (GO:0050727)**	378	8	.73	+	10.90	6.36E-07	6.03 E-05
**protein autophosphorylation (GO:0046777)**	156	6	.30	+	19.80	6.57E-07	6.19 E-05
**response to inorganic substance (GO:0010035)**	528	9	1.03	+	8.78	6.83E-07	6.39 E-05
**blood vessel development (GO:0001568)**	529	9	1.03	+	8.76	6.94E-07	6.45 E-05
**regulation of cell cycle (GO:0051726)**	1094	12	2.13	+	5.65	7.33E-07	6.78 E-05
**regulation of cell differentiation (GO:0045595)**	1563	14	3.04	+	4.61	7.64E-07	7.03 E-05
**peptidyl-lysine deacetylation (GO:0034983)**	7	3	.01	+	> 100	8.02E-07	7.33 E-05
**negative regulation of cellular component organization (GO:0051129)**	708	10	1.38	+	7.27	8.05E-07	7.32 E-05
**positive regulation of smooth muscle cell proliferation (GO:0048661)**	85	5	.17	+	30.28	8.23E-07	7.44 E-05
**positive regulation of response to external stimulus (GO:0032103)**	548	9	1.06	+	8.45	9.26E-07	8.31 E-05
**vasculature development (GO:0001944)**	550	9	1.07	+	8.42	9.53E-07	8.51 E-05
**negative regulation of programmed cell death (GO:0043069)**	923	11	1.79	+	6.14	1.07E-06	9.46 E-05
**circulatory system development (GO:0072359)**	926	11	1.80	+	6.12	1.10E-06	9.71 E-05
**regulation of leukocyte activation (GO:0002694)**	566	9	1.10	+	8.19	1.20E-06	1.06 E-04
**cellular response to stress (GO:0033554)**	1630	14	3.17	+	4.42	1.26E-06	1.10 E-04

**response to angiotensin (GO:1990776)**	38	4	.07	+	54.19	1.29E-06	1.11 E-04
**cellular response to chemical stress (GO:0062197)**	285	7	.55	+	12.64	1.32E-06	1.14 E-04
**chromatin organization (GO:0006325)**	751	10	1.46	+	6.85	1.36E-06	1.17 E-04
**response to reactive oxygen species (GO:0000302)**	179	6	.35	+	17.26	1.43E-06	1.22 E-04
**cellular response to organonitrogen compound (GO:0071417)**	587	9	1.14	+	7.89	1.62E-06	1.37 E-04
**response to hydrogen peroxide (GO:0042542)**	99	5	.19	+	26.00	1.69E-06	1.42 E-04
**regulation of G2/M transition of mitotic cell cycle (GO:0010389)**	101	5	.20	+	25.49	1.86E-06	1.56 E-04
**positive regulation of protein import into nucleus (GO:0042307)**	42	4	.08	+	49.03	1.86E-06	1.55 E-04
**epigenetic regulation of gene expression (GO:0040029)**	189	6	.37	+	16.34	1.94E-06	1.61 E-04
**chromatin remodeling (GO:0006338)**	602	9	1.17	+	7.70	1.99E-06	1.64 E-04
**positive regulation of endothelial cell migration (GO:0010595)**	106	5	.21	+	24.28	2.33E-06	1.91 E-04
**cellular response to ketone (GO:1901655)**	107	5	.21	+	24.06	2.44E-06	1.99 E-04
**negative regulation of metabolic process (GO:0009892)**	2902	18	5.64	+	3.19	2.45E-06	1.99 E-04
**regulation of cell activation (GO:0050865)**	623	9	1.21	+	7.44	2.62E-06	2.12 E-04
**positive regulation of protein-containing complex assembly (GO:0031334)**	200	6	.39	+	15.44	2.66E-06	2.14 E-04
**negative regulation of gene expression, epigenetic (GO:0045814)**	110	5	.21	+	23.40	2.78E-06	2.22 E-04
**regulation of catabolic process (GO:0009894)**	1026	11	1.99	+	5.52	2.95E-06	2.35 E-04
**response to amine (GO:0014075)**	48	4	.09	+	42.90	3.07E-06	2.43 E-04
**regulation of cell cycle G2/M phase transition (GO:1902749)**	114	5	.22	+	22.58	3.28E-06	2.59 E-04
**regulation of protein kinase activity (GO:0045859)**	473	8	.92	+	8.71	3.29E-06	2.57 E-04

**tissue development (GO:0009888)**	1768	14	3.43	+	4.08	3.29E-06	2.56 E-04
**cell differentiation (GO:0030154)**	3648	20	7.09	+	2.82	3.33E-06	2.59 E-04
**cellular developmental process (GO:0048869)**	3651	20	7.09	+	2.82	3.38E-06	2.61 E-04
**cellular response to growth factor stimulus (GO:0071363)**	475	8	.92	+	8.67	3.39E-06	2.60 E-04
**cellular process (GO:0009987)**	14924	40	28.99	+	1.38	3.40E-06	2.59 E-04
**negative regulation of cellular metabolic process (GO:0031324)**	2648	17	5.14	+	3.30	3.41E-06	2.59 E-04
**regulation of nucleocytoplasmic transport (GO:0046822)**	116	5	.23	+	22.19	3.56E-06	2.70 E-04
**G2/M transition of mitotic cell cycle (GO:0000086)**	50	4	.10	+	41.18	3.57E-06	2.69 E-04
**protein-DNA complex organization (GO:0071824)**	839	10	1.63	+	6.14	3.64E-06	2.72 E-04
**response to oxygen levels (GO:0070482)**	336	7	.65	+	10.72	3.84E-06	2.86 E-04
**protein destabilization (GO:0031648)**	51	4	.10	+	40.38	3.85E-06	2.85 E-04
**regulation of plasma membrane bounded cell projection organization (GO:0120035)**	654	9	1.27	+	7.08	3.88E-06	2.86 E-04
**positive regulation of mitotic cell cycle (GO:0045931)**	120	5	.23	+	21.45	4.18E-06	3.07 E-04
**regulation of cell-matrix adhesion (GO:0001952)**	122	5	.24	+	21.10	4.52E-06	3.30 E-04
**positive regulation of protein localization (GO:1903829)**	496	8	.96	+	8.30	4.64E-06	3.37 E-04
**regulation of cell projection organization (GO:0031344)**	669	9	1.30	+	6.93	4.65E-06	3.37 E-04
**cell population proliferation (GO:0008283)**	1823	14	3.54	+	3.95	4.70E-06	3.39 E-04
**cell cycle G2/M phase transition (GO:0044839)**	55	4	.11	+	37.44	5.10E-06	3.66 E-04
**positive regulation of organelle organization (GO:0010638)**	505	8	.98	+	8.16	5.28E-06	3.77 E-04
**sphingosine-1-phosphate receptor signaling pathway (GO:0003376)**	15	3	.03	+	> 100	5.40E-06	3.84 E-04
**regulation of chemotaxis (GO:0050920)**	228	6	.44	+	13.55	5.55E-06	3.93 E-04

**tube morphogenesis (GO:0035239)**	684	9	1.33	+	6.77	5.55E-06	3.91 E-04
**mitotic cell cycle process (GO:1903047)**	509	8	.99	+	8.09	5.59E-06	3.92 E-04
**positive regulation of cell projection organization (GO:0031346)**	358	7	.70	+	10.07	5.77E-06	4.03 E-04
**cellular response to oxidative stress (GO:0034599)**	230	6	.45	+	13.43	5.83E-06	4.05 E-04
**cellular response to heat (GO:0034605)**	57	4	.11	+	36.13	5.84E-06	4.04 E-04
**regulation of protein localization (GO:0032880)**	892	10	1.73	+	5.77	6.22E-06	4.28 E-04
**sphingolipid mediated signaling pathway (GO:0090520)**	16	3	.03	+	96.53	6.40E-06	4.39 E-04
**negative regulation of apoptotic process (GO:0043066)**	896	10	1.74	+	5.75	6.47E-06	4.41 E-04
**cell migration (GO:0016477)**	899	10	1.75	+	5.73	6.66E-06	4.52 E-04
**regulation of protein import into nucleus (GO:0042306)**	61	4	.12	+	33.76	7.53E-06	5.09 E-04
**regulation of smooth muscle cell proliferation (GO:0048660)**	139	5	.27	+	18.52	8.35E-06	5.62 E-04
**regulation of cell cycle process (GO:0010564)**	726	9	1.41	+	6.38	8.92E-06	5.98 E-04
**regulation of muscle cell differentiation (GO:0051147)**	142	5	.28	+	18.13	9.23E-06	6.16 E-04
**regulation of system process (GO:0044057)**	547	8	1.06	+	7.53	9.38E-06	6.24 E-04
**negative regulation of molecular function (GO:0044092)**	736	9	1.43	+	6.30	9.95E-06	6.58 E-04
**mitotic cell cycle phase transition (GO:0044772)**	145	5	.28	+	17.75	1.02E-05	6.71 E-04
**positive regulation of cell cycle process (GO:0090068)**	256	6	.50	+	12.07	1.06E-05	6.94 E-04
**regulation of organelle organization (GO:0033043)**	1181	11	2.29	+	4.79	1.12E-05	7.31 E-04
**cellular response to fluid shear stress (GO:0071498)**	20	3	.04	+	77.22	1.16E-05	7.57 E-04
**protein-containing complex organization (GO:0043933)**	2006	14	3.90	+	3.59	1.41E-05	9.16 E-04
**regulation of defense response (GO:0031347)**	774	9	1.50	+	5.99	1.48E-05	9.55 E-04

**cell cycle phase transition (GO:0044770)**	158	5	.31	+	16.29	1.52E-05	9.78 E-04
**regulation of protein-containing complex assembly (GO:0043254)**	421	7	.82	+	8.56	1.62E-05	1.04 E-03
**regulation of cellular localization (GO:0060341)**	999	10	1.94	+	5.15	1.66E-05	1.06 E-03
**supramolecular fiber organization (GO:0097435)**	593	8	1.15	+	6.95	1.67E-05	1.06 E-03
**mitotic cell cycle (GO:0000278)**	594	8	1.15	+	6.93	1.69E-05	1.07 E-03
**regulation of cell adhesion (GO:0030155)**	788	9	1.53	+	5.88	1.70E-05	1.07 E-03
**positive regulation of intracellular protein transport (GO:0090316)**	162	5	.31	+	15.89	1.71E-05	1.07 E-03
**cellular component organization (GO:0016043)**	5610	24	10.90	+	2.20	1.73E-05	1.08 E-03
**cytoskeleton organization (GO:0007010)**	1256	11	2.44	+	4.51	1.98E-05	1.23 E-03
**regulation of endothelial cell migration (GO:0010594)**	168	5	.33	+	15.32	2.03E-05	1.26 E-03
**response to hypoxia (GO:0001666)**	294	6	.57	+	10.51	2.28E-05	1.40 E-03
**regulation of immune system process (GO:0002682)**	1531	12	2.97	+	4.04	2.28E-05	1.40 E-03
**mitotic DNA damage checkpoint signaling (GO:0044773)**	85	4	.17	+	24.23	2.63E-05	1.61 E-03
**regulation of striated muscle cell differentiation (GO:0051153)**	85	4	.17	+	24.23	2.63E-05	1.60 E-03
**regulation of cell development (GO:0060284)**	834	9	1.62	+	5.56	2.66E-05	1.61 E-03
**histone modification (GO:0016570)**	86	4	.17	+	23.94	2.74E-05	1.66 E-03
**positive regulation of signal transduction (GO:0009967)**	1563	12	3.04	+	3.95	2.80E-05	1.68 E-03
**response to decreased oxygen levels (GO:0036293)**	307	6	.60	+	10.06	2.89E-05	1.73 E-03
**positive regulation of phosphatidylinositol 3-kinase/protein kinase B signal transduction (GO:0051897)**	182	5	.35	+	14.14	2.95E-05	1.76 E-03
**cellular component organization or biogenesis (GO:0071840)**	5824	24	11.31	+	2.12	3.02E-05	1.79 E-03

**cell motility (GO:0048870)**	1075	10	2.09	+	4.79	3.10E-05	1.84 E-03
**mitotic DNA integrity checkpoint signaling (GO:0044774)**	89	4	.17	+	23.14	3.12E-05	1.84 E-03
**positive regulation of cell differentiation (GO:0045597)**	865	9	1.68	+	5.36	3.53E-05	2.07 E-03
**regulation of stress fiber assembly (GO:0051492)**	92	4	.18	+	22.38	3.54E-05	2.07 E-03
**positive regulation of protein transport (GO:0051222)**	319	6	.62	+	9.68	3.57E-05	2.08 E-03
**response to lipopolysaccharide (GO:0032496)**	320	6	.62	+	9.65	3.63E-05	2.11 E-03
**negative regulation of cellular biosynthetic process (GO:0031327)**	2189	14	4.25	+	3.29	3.78E-05	2.19 E-03
**positive regulation of peptidyl-serine phosphorylation (GO:0033138)**	94	4	.18	+	21.91	3.84E-05	2.21 E-03
**negative regulation of cell junction assembly (GO:1901889)**	31	3	.06	+	49.82	3.87E-05	2.23 E-03
**positive regulation of signaling receptor activity (GO:2000273)**	31	3	.06	+	49.82	3.87E-05	2.22 E-03
**response to heat (GO:0009408)**	95	4	.18	+	21.68	3.99E-05	2.28 E-03
**nervous system development (GO:0007399)**	2200	14	4.27	+	3.28	4.00E-05	2.27 E-03
**negative regulation of biosynthetic process (GO:0009890)**	2203	14	4.28	+	3.27	4.06E-05	2.30 E-03
**response to alkaloid (GO:0043279)**	96	4	.19	+	21.45	4.15E-05	2.34 E-03
**cell cycle process (GO:0022402)**	884	9	1.72	+	5.24	4.17E-05	2.35 E-03
**response to amphetamine (GO:0001975)**	32	3	.06	+	48.26	4.23E-05	2.37 E-03
**regulation of mitotic cell cycle phase transition (GO:1901990)**	332	6	.64	+	9.30	4.44E-05	2.48 E-03
**regulation of protein stability (GO:0031647)**	334	6	.65	+	9.25	4.58E-05	2.55 E-03
**positive regulation of establishment of protein localization (GO:1904951)**	335	6	.65	+	9.22	4.66E-05	2.58 E-03

**negative regulation of locomotion (GO:0040013)**	336	6	.65	+	9.19	4.74E-05	2.62 E-03
**response to molecule of bacterial origin (GO:0002237)**	338	6	.66	+	9.14	4.89E-05	2.69 E-03
**tube development (GO:0035295)**	904	9	1.76	+	5.13	4.96E-05	2.72 E-03
**regulation of neuroinflammatory response (GO:0150077)**	34	3	.07	+	45.42	5.01E-05	2.74 E-03
**response to fluid shear stress (GO:0034405)**	34	3	.07	+	45.42	5.01E-05	2.73 E-03
**angiogenesis (GO:0001525)**	340	6	.66	+	9.08	5.05E-05	2.74 E-03
**negative regulation of response to stimulus (GO:0048585)**	1662	12	3.23	+	3.72	5.11E-05	2.76 E-03
**positive regulation of intracellular transport (GO:0032388)**	205	5	.40	+	12.56	5.13E-05	2.76 E-03
**regulation of actomyosin structure organization (GO:0110020)**	102	4	.20	+	20.19	5.22E-05	2.80 E-03
**positive regulation of tumor necrosis factor production (GO:0032760)**	102	4	.20	+	20.19	5.22E-05	2.79 E-03
**positive regulation of fibroblast growth factor production (GO:0090271)**	4	2	.01	+	> 100	5.47E-05	2.92 E-03
**regulation of intracellular transport (GO:0032386)**	345	6	.67	+	8.95	5.47E-05	2.91 E-03
**regulation of actin filament bundle assembly (GO:0032231)**	104	4	.20	+	19.80	5.61E-05	2.97 E-03
**regulation of cell junction assembly (GO:1901888)**	211	5	.41	+	12.20	5.86E-05	3.09 E-03
**positive regulation of lamellipodium organization (GO:1902745)**	36	3	.07	+	42.90	5.88E-05	3.09 E-03
**negative regulation of JNK cascade (GO:0046329)**	36	3	.07	+	42.90	5.88E-05	3.08 E-03
**regulation of cytoskeleton organization (GO:0051493)**	518	7	1.01	+	6.96	5.97E-05	3.12 E-03
**positive regulation of tumor necrosis factor superfamily cytokine production (GO:1903557)**	106	4	.21	+	19.43	6.03E-05	3.14 E-03
**positive regulation of vascular associated smooth**	37	3	.07	+	41.74	6.35E-05	3.29 E-03

**muscle cell proliferation (GO:1904707)**							
**platelet-derived growth factor receptor signaling pathway (GO:0048008)**	37	3	.07	+	41.74	6.35E-05	3.28 E-03
**negative regulation of nitrogen compound metabolic process (GO:0051172)**	2294	14	4.46	+	3.14	6.35E-05	3.27 E-03
**membrane protein proteolysis (GO:0033619)**	38	3	.07	+	40.64	6.84E-05	3.51 E-03
**positive regulation of leukocyte activation (GO:0002696)**	360	6	.70	+	8.58	6.90E-05	3.53 E-03
**cell activation (GO:0001775)**	731	8	1.42	+	5.63	7.26E-05	3.70 E-03
**response to antibiotic (GO:0046677)**	39	3	.08	+	39.60	7.35E-05	3.74 E-03
**positive regulation of smooth muscle cell migration (GO:0014911)**	39	3	.08	+	39.60	7.35E-05	3.73 E-03
**DNA damage checkpoint signaling (GO:0000077)**	112	4	.22	+	18.39	7.42E-05	3.74 E-03
**regulation of anaphase-promoting complex-dependent catabolic process (GO:1905784)**	5	2	.01	+	> 100	7.65E-05	3.85 E-03
**positive regulation of nitric oxide biosynthetic process (GO:0045429)**	40	3	.08	+	38.61	7.89E-05	3.96 E-03
**regulation of establishment of protein localization (GO:0070201)**	542	7	1.05	+	6.65	7.91E-05	3.95 E-03
**actin cytoskeleton organization (GO:0030036)**	543	7	1.05	+	6.64	8.00E-05	3.99 E-03
**anatomical structure formation involved in morphogenesis (GO:0048646)**	964	9	1.87	+	4.81	8.11E-05	4.03 E-03
**regulation of muscle system process (GO:0090257)**	228	5	.44	+	11.29	8.39E-05	4.15 E-03
**collagen catabolic process (GO:0030574)**	41	3	.08	+	37.67	8.46E-05	4.18 E-03
**positive regulation of nitric oxide metabolic process (GO:1904407)**	41	3	.08	+	37.67	8.46E-05	4.16 E-03
**negative regulation of intracellular signal transduction (GO:1902532)**	548	7	1.06	+	6.58	8.46E-05	4.15 E-03

**response to salt (GO:1902074)**	374	6	.73	+	8.26	8.48E-05	4.15 E-03
**regulation of DNA biosynthetic process (GO:2000278)**	117	4	.23	+	17.60	8.74E-05	4.26 E-03
**actomyosin structure organization (GO:0031032)**	117	4	.23	+	17.60	8.74E-05	4.24 E-03
**positive regulation of cell activation (GO:0050867)**	378	6	.73	+	8.17	8.99E-05	4.35 E-03
**regulation of mitotic nuclear division (GO:0007088)**	118	4	.23	+	17.45	9.02E-05	4.35 E-03
**regulation of hydrolase activity (GO:0051336)**	755	8	1.47	+	5.45	9.07E-05	4.36 E-03
**cellular response to biotic stimulus (GO:0071216)**	233	5	.45	+	11.05	9.27E-05	4.45 E-03
**regulation of intracellular protein transport (GO:0033157)**	234	5	.45	+	11.00	9.45E-05	4.52 E-03
**negative regulation of gene expression (GO:0010629)**	985	9	1.91	+	4.70	9.55E-05	4.55 E-03
**regulation of myotube differentiation (GO:0010830)**	43	3	.08	+	35.92	9.67E-05	4.60 E-03
**regulation of inflammatory response to antigenic stimulus (GO:0002861)**	43	3	.08	+	35.92	9.67E-05	4.58 E-03
**regulation of RNA metabolic process (GO:0051252)**	3773	18	7.33	+	2.46	9.76E-05	4.61 E-03
**DNA integrity checkpoint signaling (GO:0031570)**	121	4	.24	+	17.02	9.91E-05	4.67 E-03
**positive regulation of immune system process (GO:0002684)**	995	9	1.93	+	4.66	1.03E-04	4.84 E-03
**cell surface receptor signaling pathway (GO:0007166)**	2092	13	4.06	+	3.20	1.07E-04	4.99 E-03
**negative regulation of transcription by RNA polymerase II (GO:0000122)**	1000	9	1.94	+	4.63	1.07E-04	5.00 E-03
**regulation of protein modification by small protein conjugation or removal (GO:1903320)**	241	5	.47	+	10.68	1.08E-04	5.04 E-03
**cellular response to vascular endothelial growth factor stimulus (GO:0035924)**	45	3	.09	+	34.32	1.10E-04	5.10 E-03
**positive regulation of muscle contraction (GO:0045933)**	45	3	.09	+	34.32	1.10E-04	5.08 E-03
**tissue morphogenesis (GO:0048729)**	572	7	1.11	+	6.30	1.10E-04	5.08 E-03

**regulation of peptidyl-serine phosphorylation (GO:0033135)**	126	4	.24	+	16.34	1.15E-04	5.30 E-03
**substrate adhesion-dependent cell spreading (GO:0034446)**	46	3	.09	+	33.57	1.17E-04	5.36 E-03
**regulation of DNA-templated transcription (GO:0006355)**	3459	17	6.72	+	2.53	1.19E-04	5.42 E-03
**cellular response to peptide hormone stimulus (GO:0071375)**	246	5	.48	+	10.46	1.19E-04	5.42 E-03
**cell cycle (GO:0007049)**	1265	10	2.46	+	4.07	1.21E-04	5.48 E-03
**negative regulation of stress-activated MAPK cascade (GO:0032873)**	47	3	.09	+	32.86	1.24E-04	5.63 E-03
**extracellular matrix disassembly (GO:0022617)**	47	3	.09	+	32.86	1.24E-04	5.61 E-03
**regulation of RNA biosynthetic process (GO:2001141)**	3477	17	6.75	+	2.52	1.27E-04	5.71 E-03
**regulation of phosphatidylinositol 3-kinase/protein kinase B signal transduction (GO:0051896)**	250	5	.49	+	10.30	1.28E-04	5.75 E-03
**mitotic cell cycle checkpoint signaling (GO:0007093)**	130	4	.25	+	15.84	1.30E-04	5.80 E-03
**interleukin-3-mediated signaling pathway (GO:0038156)**	7	2	.01	+	> 100	1.31E-04	5.84 E-03
**regulation of fibroblast growth factor production (GO:0090270)**	7	2	.01	+	> 100	1.31E-04	5.82 E-03
**negative regulation of stress-activated protein kinase signaling cascade (GO:0070303)**	48	3	.09	+	32.17	1.32E-04	5.85 E-03
**negative regulation of macromolecule biosynthetic process (GO:0010558)**	2138	13	4.15	+	3.13	1.33E-04	5.88 E-03
**regulation of blood circulation (GO:1903522)**	252	5	.49	+	10.21	1.33E-04	5.86 E-03
**positive regulation of protein modification by small protein conjugation or removal (GO:1903322)**	133	4	.26	+	15.48	1.41E-04	6.21 E-03
**biological process involved in interspecies interaction**	1562	11	3.03	+	3.63	1.43E-04	6.26 E-03

**between organisms (GO:0044419)**							
**response to alcohol (GO:0097305)**	260	5	.51	+	9.90	1.53E-04	6.71 E-03
**actin filament-based process (GO:0030029)**	605	7	1.18	+	5.96	1.55E-04	6.77 E-03
**regulation of lamellipodium organization (GO:1902743)**	51	3	.10	+	30.28	1.56E-04	6.80 E-03
**response to cocaine (GO:0042220)**	51	3	.10	+	30.28	1.56E-04	6.78 E-03
**positive regulation of protein kinase activity (GO:0045860)**	263	5	.51	+	9.79	1.62E-04	6.99 E-03
**hepatocyte growth factor receptor signaling pathway (GO:0048012)**	8	2	.02	+	> 100	1.63E-04	7.04 E-03
**negative regulation of nucleobase-containing compound metabolic process (GO:0045934)**	1587	11	3.08	+	3.57	1.64E-04	7.06 E-03
**positive regulation of stress fiber assembly (GO:0051496)**	52	3	.10	+	29.70	1.65E-04	7.08 E-03
**regulation of biological quality (GO:0065008)**	2846	15	5.53	+	2.71	1.69E-04	7.22 E-03
**regulation of microtubule-based process (GO:0032886)**	266	5	.52	+	9.68	1.70E-04	7.26 E-03
**positive regulation of synaptic transmission (GO:0050806)**	141	4	.27	+	14.60	1.76E-04	7.47 E-03
**rhythmic process (GO:0048511)**	268	5	.52	+	9.60	1.76E-04	7.47 E-03
**blood vessel morphogenesis (GO:0048514)**	431	6	.84	+	7.17	1.82E-04	7.69 E-03
**regulation of protein serine/threonine kinase activity (GO:0071900)**	270	5	.52	+	9.53	1.82E-04	7.69 E-03
**regulation of cell cycle phase transition (GO:1901987)**	434	6	.84	+	7.12	1.89E-04	7.94 E-03
**signal transduction in response to DNA damage (GO:0042770)**	145	4	.28	+	14.20	1.95E-04	8.18 E-03
**cellular response to phorbol 13-acetate 12-myristate (GO:1904628)**	9	2	.02	+	> 100	1.99E-04	8.34 E-03
**response to phorbol 13-acetate 12-myristate (GO:1904627)**	9	2	.02	+	> 100	1.99E-04	8.32 E-03
**neurogenesis (GO:0022008)**	1348	10	2.62	+	3.82	2.03E-04	8.43 E-03

**regulation of nuclear division (GO:0051783)**	147	4	.29	+	14.01	2.05E-04	8.52 E-03
**regulation of endopeptidase activity (GO:0052548)**	278	5	.54	+	9.26	2.08E-04	8.61 E-03
**cell development (GO:0048468)**	2237	13	4.35	+	2.99	2.10E-04	8.65 E-03
**response to purine-containing compound (GO:0014074)**	148	4	.29	+	13.91	2.11E-04	8.66 E-03
**response to fatty acid (GO:0070542)**	57	3	.11	+	27.09	2.14E-04	8.78 E-03
**actin filament organization (GO:0007015)**	280	5	.54	+	9.19	2.15E-04	8.81 E-03
**response to steroid hormone (GO:0048545)**	281	5	.55	+	9.16	2.19E-04	8.93 E-03
**positive regulation of cell development (GO:0010720)**	450	6	.87	+	6.86	2.29E-04	9.32 E-03
**epithelium development (GO:0060429)**	1107	9	2.15	+	4.19	2.29E-04	9.31 E-03
**regulation of post-translational protein modification (GO:1901873)**	285	5	.55	+	9.03	2.33E-04	9.44 E-03
**positive regulation of immune response (GO:0050778)**	648	7	1.26	+	5.56	2.36E-04	9.52 E-03
**negative regulation of cell migration (GO:0030336)**	286	5	.56	+	9.00	2.37E-04	9.54 E-03
**negative regulation of protein acetylation (GO:1901984)**	10	2	.02	+	> 100	2.39E-04	9.60 E-03
**response to other organism (GO:0051707)**	1379	10	2.68	+	3.73	2.43E-04	9.75 E-03
**response to corticosteroid (GO:0031960)**	154	4	.30	+	13.37	2.44E-04	9.76 E-03
**positive regulation of endopeptidase activity (GO:0010950)**	154	4	.30	+	13.37	2.44E-04	9.73 E-03
**regulation of anatomical structure morphogenesis (GO:0022603)**	874	8	1.70	+	4.71	2.46E-04	9.78 E-03
**regulation of vascular associated smooth muscle cell proliferation (GO:1904705)**	60	3	.12	+	25.74	2.47E-04	9.81 E-03
**response to external biotic stimulus (GO:0043207)**	1382	10	2.68	+	3.73	2.48E-04	9.80 E-03
**regulation of neuron projection development (GO:0010975)**	458	6	.89	+	6.74	2.51E-04	9.92 E-03
**morphogenesis of an epithelium (GO:0002009)**	460	6	.89	+	6.71	2.57E-04	1.01 E-02

**ossification (GO:0001503)**	293	5	.57	+	8.78	2.64E-04	1.04 E-02
**collagen metabolic process (GO:0032963)**	62	3	.12	+	24.91	2.71E-04	1.06 E-02
**negative regulation of chemotaxis (GO:0050922)**	62	3	.12	+	24.91	2.71E-04	1.06 E-02
**positive regulation of actin filament bundle assembly (GO:0032233)**	62	3	.12	+	24.91	2.71E-04	1.06 E-02
**phagocytosis (GO:0006909)**	159	4	.31	+	12.95	2.75E-04	1.07 E-02
**regulation of ERK1 and ERK2 cascade (GO:0070372)**	296	5	.57	+	8.70	2.77E-04	1.07 E-02
**negative regulation of fibroblast migration (GO:0010764)**	11	2	.02	+	93.60	2.82E-04	1.09 E-02
**regulation of peptidase activity (GO:0052547)**	301	5	.58	+	8.55	2.99E-04	1.15 E-02
**negative regulation of cell motility (GO:2000146)**	301	5	.58	+	8.55	2.99E-04	1.15 E-02
**regulation of muscle contraction (GO:0006937)**	163	4	.32	+	12.63	3.01E-04	1.16 E-02
**regulation of microtubule cytoskeleton organization (GO:0070507)**	163	4	.32	+	12.63	3.01E-04	1.15 E-02
**positive regulation of post-translational protein modification (GO:1901875)**	164	4	.32	+	12.56	3.08E-04	1.18 E-02
**cell cycle checkpoint signaling (GO:0000075)**	164	4	.32	+	12.56	3.08E-04	1.17 E-02
**regulation of smooth muscle cell migration (GO:0014910)**	65	3	.13	+	23.76	3.10E-04	1.18 E-02
**regulation of tumor necrosis factor production (GO:0032680)**	165	4	.32	+	12.48	3.15E-04	1.20 E-02
**positive regulation of peptidyl-tyrosine phosphorylation (GO:0050731)**	165	4	.32	+	12.48	3.15E-04	1.19 E-02
**regulation of cysteine-type endopeptidase activity involved in apoptotic process (GO:0043281)**	165	4	.32	+	12.48	3.15E-04	1.19 E-02
**regulation of immune response (GO:0050776)**	908	8	1.76	+	4.54	3.17E-04	1.19 E-02
**response to biotic stimulus (GO:0009607)**	1426	10	2.77	+	3.61	3.18E-04	1.20 E-02

**cellular response to peptide (GO:1901653)**	306	5	.59	+	8.41	3.22E-04	1.20 E-02
**G1/S transition of mitotic cell cycle (GO:0000082)**	66	3	.13	+	23.40	3.24E-04	1.21 E-02
**regulation of ceramide biosynthetic process (GO:2000303)**	12	2	.02	+	85.80	3.29E-04	1.22 E-02
**regulation of sphingolipid biosynthetic process (GO:0090153)**	12	2	.02	+	85.80	3.29E-04	1.22 E-02
**regulation of membrane lipid metabolic process (GO:1905038)**	12	2	.02	+	85.80	3.29E-04	1.22 E-02
**response to temperature stimulus (GO:0009266)**	167	4	.32	+	12.33	3.30E-04	1.22 E-02
**response to bacterium (GO:0009617)**	687	7	1.33	+	5.25	3.35E-04	1.24 E-02
**negative regulation of mitotic cell cycle phase transition (GO:1901991)**	168	4	.33	+	12.26	3.37E-04	1.24 E-02
**positive regulation of peptidase activity (GO:0010952)**	168	4	.33	+	12.26	3.37E-04	1.24 E-02
**regulation of protein processing (GO:0070613)**	67	3	.13	+	23.05	3.38E-04	1.24 E-02
**positive regulation of cell adhesion (GO:0045785)**	485	6	.94	+	6.37	3.40E-04	1.24 E-02
**negative regulation of macromolecule metabolic process (GO:0010605)**	2685	14	5.22	+	2.68	3.43E-04	1.25 E-02
**generation of neurons (GO:0048699)**	1172	9	2.28	+	3.95	3.49E-04	1.27 E-02
**cell cycle G1/S phase transition (GO:0044843)**	68	3	.13	+	22.71	3.52E-04	1.28 E-02
**regulation of tumor necrosis factor superfamily cytokine production (GO:1903555)**	170	4	.33	+	12.11	3.52E-04	1.28 E-02
**peptidyl-lysine modification (GO:0018205)**	171	4	.33	+	12.04	3.60E-04	1.30 E-02
**hemopoiesis (GO:0030097)**	696	7	1.35	+	5.18	3.62E-04	1.30 E-02
**regulation of lipid metabolic process (GO:0019216)**	315	5	.61	+	8.17	3.67E-04	1.32 E-02
**positive regulation of hydrolase activity (GO:0051345)**	494	6	.96	+	6.25	3.75E-04	1.34 E-02
**positive regulation of nucleobase-containing**	2052	12	3.99	+	3.01	3.76E-04	1.35 E-02

**compound metabolic process (GO:0045935)**							
**positive regulation of cell size (GO:0045793)**	13	2	.03	+	79.20	3.79E-04	1.35 E-02
**regulation of skeletal muscle fiber development (GO:0048742)**	13	2	.03	+	79.20	3.79E-04	1.35 E-02
**negative regulation of transcription by competitive promoter binding (GO:0010944)**	13	2	.03	+	79.20	3.79E-04	1.35 E-02
**positive regulation of supramolecular fiber organization (GO:1902905)**	175	4	.34	+	11.77	3.92E-04	1.39 E-02
**response to estrogen (GO:0043627)**	71	3	.14	+	21.75	3.98E-04	1.41 E-02
**cellular response to abiotic stimulus (GO:0071214)**	323	5	.63	+	7.97	4.11E-04	1.45 E-02
**cellular response to environmental stimulus (GO:0104004)**	323	5	.63	+	7.97	4.11E-04	1.45 E-02
**cellular response to hormone stimulus (GO:0032870)**	503	6	.98	+	6.14	4.12E-04	1.45 E-02
**regulation of lymphocyte activation (GO:0051249)**	503	6	.98	+	6.14	4.12E-04	1.44 E-02
**positive regulation of TOR signaling (GO:0032008)**	72	3	.14	+	21.45	4.14E-04	1.45 E-02
**regulation of protein maturation (GO:1903317)**	72	3	.14	+	21.45	4.14E-04	1.44 E-02
**post-translational protein modification (GO:0043687)**	946	8	1.84	+	4.35	4.17E-04	1.45 E-02
**interleukin-6-mediated signaling pathway (GO:0070102)**	14	2	.03	+	73.54	4.32E-04	1.50 E-02
**sphingoid biosynthetic process (GO:0046520)**	14	2	.03	+	73.54	4.32E-04	1.50 E-02
**sphingosine biosynthetic process (GO:0046512)**	14	2	.03	+	73.54	4.32E-04	1.49 E-02
**regulation of small molecule metabolic process (GO:0062012)**	327	5	.64	+	7.87	4.34E-04	1.50 E-02
**regulation of intracellular steroid hormone receptor signaling pathway (GO:0033143)**	74	3	.14	+	20.87	4.47E-04	1.54 E-02
**cellular component disassembly (GO:0022411)**	330	5	.64	+	7.80	4.52E-04	1.55 E-02

**positive regulation of cytoskeleton organization (GO:0051495)**	184	4	.36	+	11.19	4.72E-04	1.62 E-02
**regulation of protein transport (GO:0051223)**	517	6	1.00	+	5.97	4.76E-04	1.63 E-02
**cellular response to nitric oxide (GO:0071732)**	15	2	.03	+	68.64	4.89E-04	1.67 E-02
**angiotensin-activated signaling pathway (GO:0038166)**	15	2	.03	+	68.64	4.89E-04	1.67 E-02
**collagen-activated signaling pathway (GO:0038065)**	15	2	.03	+	68.64	4.89E-04	1.66 E-02
**positive regulation of mast cell degranulation (GO:0043306)**	15	2	.03	+	68.64	4.89E-04	1.66 E-02
**regulation of anatomical structure size (GO:0090066)**	520	6	1.01	+	5.94	4.90E-04	1.66 E-02
**regulation of actin cytoskeleton organization (GO:0032956)**	337	5	.65	+	7.64	4.97E-04	1.68 E-02
**regulation of tissue remodeling (GO:0034103)**	77	3	.15	+	20.06	5.00E-04	1.68 E-02
**regulation of DNA metabolic process (GO:0051052)**	526	6	1.02	+	5.87	5.21E-04	1.75 E-02
**regulation of stress-activated MAPK cascade (GO:0032872)**	189	4	.37	+	10.90	5.21E-04	1.75 E-02
**negative regulation of organelle organization (GO:0010639)**	342	5	.66	+	7.53	5.30E-04	1.77 E-02
**regulation of vesicle-mediated transport (GO:0060627)**	529	6	1.03	+	5.84	5.36E-04	1.79 E-02
**positive regulation of programmed cell death (GO:0043068)**	530	6	1.03	+	5.83	5.42E-04	1.80 E-02
**response to ether (GO:0045472)**	16	2	.03	+	64.35	5.50E-04	1.83 E-02
**negative regulation of focal adhesion assembly (GO:0051895)**	16	2	.03	+	64.35	5.50E-04	1.82 E-02
**positive regulation of calcium ion import (GO:0090280)**	16	2	.03	+	64.35	5.50E-04	1.82 E-02
**response to caffeine (GO:0031000)**	16	2	.03	+	64.35	5.50E-04	1.82 E-02
**negative regulation of cell-substrate junction organization (GO:0150118)**	16	2	.03	+	64.35	5.50E-04	1.81 E-02

**chemotaxis (GO:0006935)**	345	5	.67	+	7.46	5.52E-04	1.81 E-02
**regulation of stress-activated protein kinase signaling cascade (GO:0070302)**	193	4	.37	+	10.67	5.63E-04	1.85 E-02
**taxis (GO:0042330)**	347	5	.67	+	7.42	5.66E-04	1.85 E-02
**animal organ morphogenesis (GO:0009887)**	994	8	1.93	+	4.14	5.78E-04	1.89 E-02
**regulation of cysteine-type endopeptidase activity (GO:2000116)**	195	4	.38	+	10.56	5.85E-04	1.91 E-02
**regulation of cell size (GO:0008361)**	195	4	.38	+	10.56	5.85E-04	1.90 E-02
**cellular response to lipopolysaccharide (GO:0071222)**	196	4	.38	+	10.51	5.96E-04	1.93 E-02
**positive regulation of B cell differentiation (GO:0045579)**	17	2	.03	+	60.56	6.14E-04	1.99 E-02
**cellular response to reactive nitrogen species (GO:1902170)**	17	2	.03	+	60.56	6.14E-04	1.98 E-02
**response to vitamin (GO:0033273)**	83	3	.16	+	18.61	6.18E-04	1.99 E-02
**regulation of protein ubiquitination (GO:0031396)**	198	4	.38	+	10.40	6.19E-04	1.99 E-02
**regulation of heart contraction (GO:0008016)**	199	4	.39	+	10.35	6.30E-04	2.02 E-02
**regulation of hydrogen peroxide metabolic process (GO:0010310)**	18	2	.03	+	57.20	6.81E-04	2.18 E-02
**negative regulation of glycolytic process (GO:0045820)**	18	2	.03	+	57.20	6.81E-04	2.18 E-02
**inflammatory response (GO:0006954)**	554	6	1.08	+	5.58	6.82E-04	2.18 E-02
**cellular response to salt (GO:1902075)**	204	4	.40	+	10.09	6.90E-04	2.20 E-02
**negative regulation of cell cycle (GO:0045786)**	363	5	.71	+	7.09	6.92E-04	2.20 E-02
**locomotion (GO:0040011)**	367	5	.71	+	7.01	7.27E-04	2.30 E-02
**cellular response to molecule of bacterial origin (GO:0071219)**	207	4	.40	+	9.95	7.28E-04	2.31 E-02
**negative regulation of signal transduction (GO:0009968)**	1300	9	2.53	+	3.56	7.39E-04	2.33 E-02

**regulation of MHC class II biosynthetic process (GO:0045346)**	19	2	.04	+	54.19	7.52E-04	2.37 E-02
**stress fiber assembly (GO:0043149)**	19	2	.04	+	54.19	7.52E-04	2.37 E-02
**contractile actin filament bundle assembly (GO:0030038)**	19	2	.04	+	54.19	7.52E-04	2.36 E-02
**positive regulation of mitotic cell cycle phase transition (GO:1901992)**	89	3	.17	+	17.35	7.52E-04	2.36 E-02
**regulation of DNA-binding transcription factor activity (GO:0051090)**	373	5	.72	+	6.90	7.81E-04	2.44 E-02
**regulation of protein catabolic process (GO:0042176)**	373	5	.72	+	6.90	7.81E-04	2.44 E-02
**positive regulation of protein polymerization (GO:0032273)**	91	3	.18	+	16.97	8.01E-04	2.49 E-02
**cellular response to UV (GO:0034644)**	91	3	.18	+	16.97	8.01E-04	2.49 E-02
**centrosome cycle (GO:0007098)**	92	3	.18	+	16.79	8.26E-04	2.56 E-02
**positive regulation of reproductive process (GO:2000243)**	92	3	.18	+	16.79	8.26E-04	2.56 E-02
**positive regulation of bone resorption (GO:0045780)**	20	2	.04	+	51.48	8.26E-04	2.55 E-02
**immune response-regulating cell surface receptor signaling pathway involved in phagocytosis (GO:0002433)**	20	2	.04	+	51.48	8.26E-04	2.55 E-02
**response to nitric oxide (GO:0071731)**	20	2	.04	+	51.48	8.26E-04	2.54 E-02
**sphingosine metabolic process (GO:0006670)**	20	2	.04	+	51.48	8.26E-04	2.54 E-02
**Fc-gamma receptor signaling pathway involved in phagocytosis (GO:0038096)**	20	2	.04	+	51.48	8.26E-04	2.53 E-02
**cell chemotaxis (GO:0060326)**	215	4	.42	+	9.58	8.37E-04	2.56 E-02
**regulation of actin filament-based process (GO:0032970)**	379	5	.74	+	6.79	8.38E-04	2.56 E-02
**regulation of T cell activation (GO:0050863)**	379	5	.74	+	6.79	8.38E-04	2.55 E-02
**female gonad development (GO:0008585)**	93	3	.18	+	16.61	8.51E-04	2.59 E-02
**negative regulation of mitotic cell cycle (GO:0045930)**	217	4	.42	+	9.49	8.65E-04	2.63 E-02

**regulation of apoptotic signaling pathway (GO:2001233)**	382	5	.74	+	6.74	8.68E-04	2.63 E-02
**cellular response to platelet-derived growth factor stimulus (GO:0036120)**	21	2	.04	+	49.03	9.04E-04	2.73 E-02
**diol biosynthetic process (GO:0034312)**	21	2	.04	+	49.03	9.04E-04	2.73 E-02
**response to mechanical stimulus (GO:0009612)**	220	4	.43	+	9.36	9.10E-04	2.74 E-02
**regulation of microtubule polymerization or depolymerization (GO:0031110)**	96	3	.19	+	16.09	9.31E-04	2.80 E-02
**negative regulation of DNA-templated transcription (GO:0045892)**	1343	9	2.61	+	3.45	9.31E-04	2.79 E-02
**regulation of supramolecular fiber organization (GO:1902903)**	389	5	.76	+	6.62	9.40E-04	2.81 E-02
**development of primary female sexual characteristics (GO:0046545)**	97	3	.19	+	15.92	9.58E-04	2.86 E-02
**response to cytokine (GO:0034097)**	825	7	1.60	+	4.37	9.85E-04	2.93 E-02
**positive regulation of cell adhesion mediated by integrin (GO:0033630)**	22	2	.04	+	46.80	9.85E-04	2.93 E-02
**Fc receptor mediated stimulatory signaling pathway (GO:0002431)**	22	2	.04	+	46.80	9.85E-04	2.92 E-02
**stimulatory C-type lectin receptor signaling pathway (GO:0002223)**	22	2	.04	+	46.80	9.85E-04	2.92 E-02
**cellular response to lectin (GO:1990858)**	22	2	.04	+	46.80	9.85E-04	2.91 E-02
**response to lectin (GO:1990840)**	22	2	.04	+	46.80	9.85E-04	2.91 E-02
**cellular response to vitamin (GO:0071295)**	22	2	.04	+	46.80	9.85E-04	2.90 E-02
**positive regulation of cell migration involved in sprouting angiogenesis (GO:0090050)**	22	2	.04	+	46.80	9.85E-04	2.90 E-02
**sphingoid metabolic process (GO:0046519)**	22	2	.04	+	46.80	9.85E-04	2.89 E-02
**DNA biosynthetic process (GO:0071897)**	98	3	.19	+	15.76	9.86E-04	2.89 E-02

**regulation of peptidyl-tyrosine phosphorylation (GO:0050730)**	225	4	.44	+	9.15	9.88E-04	2.89 E-02
**negative regulation of RNA biosynthetic process (GO:1902679)**	1357	9	2.64	+	3.41	1.00E-03	2.92 E-02
**immune system process (GO:0002376)**	2293	12	4.45	+	2.69	1.02E-03	2.98 E-02
**negative regulation of cell cycle phase transition (GO:1901988)**	229	4	.44	+	8.99	1.05E-03	3.06 E-02
**membrane protein ectodomain proteolysis (GO:0006509)**	23	2	.04	+	44.77	1.07E-03	3.10 E-02
**response to platelet-derived growth factor (GO:0036119)**	23	2	.04	+	44.77	1.07E-03	3.10 E-02
**positive regulation of cold-induced thermogenesis (GO:0120162)**	101	3	.20	+	15.29	1.07E-03	3.10 E-02
**cellular response to inorganic substance (GO:0071241)**	231	4	.45	+	8.91	1.09E-03	3.14 E-02
**leukocyte activation (GO:0045321)**	607	6	1.18	+	5.09	1.09E-03	3.14 E-02
**neuron differentiation (GO:0030182)**	1099	8	2.13	+	3.75	1.11E-03	3.19 E-02
**negative regulation of lipid metabolic process (GO:0045833)**	103	3	.20	+	14.99	1.13E-03	3.25 E-02
**negative regulation of response to external stimulus (GO:0032102)**	406	5	.79	+	6.34	1.13E-03	3.25 E-02
**positive regulation of defense response (GO:0031349)**	407	5	.79	+	6.32	1.15E-03	3.28 E-02
**positive regulation of protein processing (GO:0010954)**	24	2	.05	+	42.90	1.16E-03	3.30 E-02
**cellular response to alcohol (GO:0097306)**	104	3	.20	+	14.85	1.17E-03	3.32 E-02
**small molecule metabolic process (GO:0044281)**	1684	10	3.27	+	3.06	1.17E-03	3.32 E-02
**negative regulation of cell communication (GO:0010648)**	1396	9	2.71	+	3.32	1.22E-03	3.47 E-02
**negative regulation of signaling (GO:0023057)**	1397	9	2.71	+	3.32	1.23E-03	3.48 E-02
**regulation of dephosphorylation (GO:0035303)**	106	3	.21	+	14.57	1.23E-03	3.48 E-02

**negative regulation of multicellular organismal process (GO:0051241)**	1117	8	2.17	+	3.69	1.23E-03	3.47 E-02
**organic cyclic compound biosynthetic process (GO:1901362)**	1119	8	2.17	+	3.68	1.24E-03	3.50 E-02
**steroid catabolic process (GO:0006706)**	25	2	.05	+	41.18	1.25E-03	3.51 E-02
**vascular endothelial growth factor signaling pathway (GO:0038084)**	25	2	.05	+	41.18	1.25E-03	3.50 E-02
**positive regulation of alpha-beta T cell proliferation (GO:0046641)**	25	2	.05	+	41.18	1.25E-03	3.50 E-02
**regulation of multicellular organismal development (GO:2000026)**	1401	9	2.72	+	3.31	1.25E-03	3.51 E-02
**negative regulation of protein modification process (GO:0031400)**	417	5	.81	+	6.17	1.28E-03	3.56 E-02
**negative regulation of intrinsic apoptotic signaling pathway (GO:2001243)**	108	3	.21	+	14.30	1.30E-03	3.61 E-02
**tissue remodeling (GO:0048771)**	108	3	.21	+	14.30	1.30E-03	3.60 E-02
**behavior (GO:0007610)**	629	6	1.22	+	4.91	1.31E-03	3.63 E-02
**microtubule organizing center organization (GO:0031023)**	109	3	.21	+	14.17	1.33E-03	3.68 E-02
**uterus development (GO:0060065)**	26	2	.05	+	39.60	1.34E-03	3.71 E-02
**positive regulation of leukocyte degranulation (GO:0043302)**	26	2	.05	+	39.60	1.34E-03	3.71 E-02
**positive regulation of protein maturation (GO:1903319)**	26	2	.05	+	39.60	1.34E-03	3.70 E-02
**positive regulation of protein ubiquitination (GO:0031398)**	110	3	.21	+	14.04	1.36E-03	3.75 E-02
**regulation of signaling receptor activity (GO:0010469)**	110	3	.21	+	14.04	1.36E-03	3.75 E-02
**negative regulation of protein metabolic process (GO:0051248)**	874	7	1.70	+	4.12	1.37E-03	3.76 E-02
**regulation of calcium ion transport (GO:0051924)**	247	4	.48	+	8.34	1.39E-03	3.79 E-02
**response to radiation (GO:0009314)**	425	5	.83	+	6.06	1.39E-03	3.79 E-02

**leukocyte migration (GO:0050900)**	248	4	.48	+	8.30	1.41E-03	3.83 E-02
**regulation of binding (GO:0051098)**	249	4	.48	+	8.27	1.43E-03	3.88 E-02
**negative regulation of cell population proliferation (GO:0008285)**	880	7	1.71	+	4.09	1.43E-03	3.88 E-02
**positive regulation of cell cycle phase transition (GO:1901989)**	112	3	.22	+	13.79	1.43E-03	3.89 E-02
**female sex differentiation (GO:0046660)**	112	3	.22	+	13.79	1.43E-03	3.88 E-02
**positive regulation of interleukin-17 production (GO:0032740)**	27	2	.05	+	38.13	1.44E-03	3.89 E-02
**leukocyte differentiation (GO:0002521)**	430	5	.84	+	5.99	1.46E-03	3.94 E-02
**regulation of interleukin-1 production (GO:0032652)**	113	3	.22	+	13.67	1.47E-03	3.96 E-02
**gland development (GO:0048732)**	431	5	.84	+	5.97	1.47E-03	3.96 E-02
**cellular response to interleukin-6 (GO:0071354)**	28	2	.05	+	36.77	1.54E-03	4.13 E-02
**response to immobilization stress (GO:0035902)**	28	2	.05	+	36.77	1.54E-03	4.13 E-02
**positive regulation of G2/M transition of mitotic cell cycle (GO:0010971)**	28	2	.05	+	36.77	1.54E-03	4.12 E-02
**positive regulation of peptidyl-threonine phosphorylation (GO:0010800)**	28	2	.05	+	36.77	1.54E-03	4.11 E-02
**positive regulation of lamellipodium assembly (GO:0010592)**	28	2	.05	+	36.77	1.54E-03	4.11 E-02
**negative regulation of inflammatory response to antigenic stimulus (GO:0002862)**	28	2	.05	+	36.77	1.54E-03	4.10 E-02
**positive regulation of cysteine-type endopeptidase activity involved in apoptotic process (GO:0043280)**	115	3	.22	+	13.43	1.54E-03	4.10 E-02
**cellular response to light stimulus (GO:0071482)**	116	3	.23	+	13.31	1.58E-03	4.19 E-02
**Fc-gamma receptor signaling pathway (GO:0038094)**	29	2	.06	+	35.50	1.64E-03	4.35 E-02

**regulation of platelet aggregation (GO:0090330)**	29	2	.06	+	35.50	1.64E-03	4.35 E-02
**regulation of androgen receptor signaling pathway (GO:0060765)**	29	2	.06	+	35.50	1.64E-03	4.34 E-02
**positive regulation of smooth muscle contraction (GO:0045987)**	29	2	.06	+	35.50	1.64E-03	4.33 E-02
**negative regulation of miRNA transcription (GO:1902894)**	29	2	.06	+	35.50	1.64E-03	4.32 E-02
**skin epidermis development (GO:0098773)**	119	3	.23	+	12.98	1.70E-03	4.46 E-02
**nucleobase-containing compound biosynthetic process (GO:0034654)**	908	7	1.76	+	3.97	1.71E-03	4.47 E-02
**response to vitamin D (GO:0033280)**	30	2	.06	+	34.32	1.75E-03	4.58 E-02
**regulation of mast cell degranulation (GO:0043304)**	30	2	.06	+	34.32	1.75E-03	4.57 E-02
**diol metabolic process (GO:0034311)**	30	2	.06	+	34.32	1.75E-03	4.57 E-02
**negative regulation of RNA metabolic process (GO:0051253)**	1472	9	2.86	+	3.15	1.77E-03	4.60 E-02
**negative regulation of catalytic activity (GO:0043086)**	451	5	.88	+	5.71	1.79E-03	4.66 E-02
**proteolysis (GO:0006508)**	1187	8	2.31	+	3.47	1.81E-03	4.69 E-02
**positive regulation of cell cycle G2/M phase transition (GO:1902751)**	31	2	.06	+	33.21	1.86E-03	4.82 E-02
**negative regulation of miRNA metabolic process (GO:2000629)**	31	2	.06	+	33.21	1.86E-03	4.81 E-02
**positive regulation of cell-substrate junction organization (GO:0150117)**	31	2	.06	+	33.21	1.86E-03	4.81 E-02
**cellular response to angiotensin (GO:1904385)**	31	2	.06	+	33.21	1.86E-03	4.80 E-02
**regulation of actin filament organization (GO:0110053)**	271	4	.53	+	7.60	1.94E-03	4.98 E-02

Analysis Type: PANTHER Overrepresentation Test (Released 20231017)
Annotation Version and Release Date: GO Ontology database DOI: 10.5281/zenodo.8436609 Released 2023-10-09
Analyzed List: upload_1 (Homo sapiens)
Reference List: Homo sapiens (all genes in database)
Test Type: FISHER
Correction: FDR
