# Supplementary material for: HDAC6 as a Prognostic Factor and Druggable Target in HER2-Positive Breast Cancer
Source: Cancers (Basel). 2024 Nov 6;16(22):3752. doi: 10.3390/cancers16223752 (PMC11591923; doi:10.3390/cancers16223752)
Supplement: Supplementary file 1 [file cancers-16-03752-s001.zip › cancers-3285752-supplementary/TABLES/SupplementaryTableS7.pdf]

GO molecular function complete	Homo sapiens - REFLIST (20592)	upload_1 (40)	upload_1 (expected)	upload_1 (over/under)	upload_1 (fold Enrichment)	upload_1 (raw P-value)	upload_1 (FDR)
catalytic activity (GO:0003824)	5691	39	11.05	+	3.53	5.32E-21	2.70E-17
catalytic activity, acting on a protein (GO:0140096)	2343	28	4.55	+	6.15	5.90E-18	1.50E-14
phosphotransferase activity, alcohol group as acceptor (GO:0016773)	691	17	1.34	+	12.67	4.46E-15	7.54E-12
kinase activity (GO:0016301)	764	17	1.48	+	11.45	2.22E-14	2.82E-11
transferase activity, transferring phosphorus-containing groups (GO:0016772)	940	18	1.83	+	9.86	3.71E-14	3.76E-11
protein kinase activity (GO:0004672)	581	15	1.13	+	13.29	1.41E-13	1.19E-10
protein tyrosine kinase activity (GO:0004713)	147	10	.29	+	35.02	3.40E-13	2.46E-10
enzyme binding (GO:0019899)	2118	22	4.11	+	5.35	3.58E-12	2.27E-09
ATP binding (GO:0005524)	1496	19	2.91	+	6.54	7.47E-12	4.21E-09
ion binding (GO:0043167)	6145	33	11.94	+	2.76	8.41E-12	4.26E-09
adenyl ribonucleotide binding (GO:0032559)	1557	19	3.02	+	6.28	1.49E-11	6.88E-09
purine ribonucleoside triphosphate binding (GO:0035639)	1846	20	3.59	+	5.58	2.84E-11	1.20E-08
adenyl nucleotide binding (GO:0030554)	1660	19	3.22	+	5.89	4.49E-11	1.75E-08
purine ribonucleotide binding (GO:0032555)	1913	20	3.72	+	5.38	5.40E-11	1.96E-08
ribonucleotide binding (GO:0032553)	1930	20	3.75	+	5.33	6.32E-11	2.14E-08
purine nucleotide binding (GO:0017076)	2017	20	3.92	+	5.10	1.39E-10	4.41E-08
nucleotide binding (GO:0000166)	2182	20	4.24	+	4.72	5.63E-10	1.68E-07
nucleoside phosphate binding (GO:1901265)	2183	20	4.24	+	4.72	5.67E-10	1.60E-07
carbohydrate derivative binding (GO:0097367)	2294	20	4.46	+	4.49	1.36E-09	3.63E-07
heterocyclic compound binding (GO:1901363)	2303	20	4.47	+	4.47	1.46E-09	3.69E-07
small molecule binding (GO:0036094)	2597	21	5.04	+	4.16	1.60E-09	3.87E-07
histone deacetylase activity (GO:0004407)	22	5	.04	+	> 100	1.65E-09	3.81E-07
transferase activity (GO:0016740)	2323	20	4.51	+	4.43	1.69E-09	3.74E-07

protein lysine deacetylase activity (GO:0033558)	23	5	.04	+	> 100	2.01E-09	4.25E-07
transmembrane receptor protein tyrosine kinase activity (GO:0004714)	62	6	.12	+	49.82	3.59E-09	7.29E-07
anion binding (GO:0043168)	2452	20	4.76	+	4.20	4.35E-09	8.49E-07
histone deacetylase binding (GO:0042826)	125	7	.24	+	28.83	5.85E-09	1.10E-06
protein kinase binding (GO:0019901)	715	12	1.39	+	8.64	7.55E-09	1.37E-06
histone modifying activity (GO:0140993)	209	8	.41	+	19.71	7.58E-09	1.33E-06
transmembrane receptor protein kinase activity (GO:0019199)	82	6	.16	+	37.67	1.73E-08	2.93E-06
deacetylase activity (GO:0019213)	38	5	.07	+	67.74	1.93E-08	3.15E-06
kinase binding (GO:0019900)	800	12	1.55	+	7.72	2.58E-08	4.08E-06
organic cyclic compound binding (GO:0097159)	6086	29	11.82	+	2.45	2.66E-08	4.09E-06
hydrolase activity, acting on carbon-nitrogen (but not peptide) bonds, in linear amides (GO:0016811)	73	5	.14	+	35.26	4.02E-07	6.00E-05
transition metal ion binding (GO:0046914)	1126	11	2.19	+	5.03	7.14E-06	1.04E-03
hydrolase activity, acting on carbon-nitrogen (but not peptide) bonds (GO:0016810)	141	5	.27	+	18.26	8.93E-06	1.26E-03
heme binding (GO:0020037)	144	5	.28	+	17.88	9.86E-06	1.35E-03
scaffold protein binding (GO:0097110)	71	4	.14	+	29.00	1.33E-05	1.78E-03
tetrapyrrole binding (GO:0046906)	154	5	.30	+	16.71	1.35E-05	1.76E-03
protein serine/threonine kinase activity (GO:0004674)	433	7	.84	+	8.32	1.94E-05	2.46E-03
histone kinase activity (GO:0035173)	25	3	.05	+	61.78	2.14E-05	2.65E-03
D-erythro-sphingosine kinase activity (GO:0017050)	2	2	.00	+	> 100	2.19E-05	2.65E-03
protein de-2-hydroxyisobutyrylase activity (GO:0160010)	2	2	.00	+	> 100	2.19E-05	2.59E-03
sphinganine kinase activity (GO:0008481)	2	2	.00	+	> 100	2.19E-05	2.53E-03
zinc ion binding (GO:0008270)	849	9	1.65	+	5.46	3.05E-05	3.44E-03
hydrolase activity (GO:0016787)	2470	15	4.80	+	3.13	3.27E-05	3.61E-03
Rho GDP-dissociation inhibitor binding (GO:0051022)	3	2	.01	+	> 100	3.65E-05	3.94E-03

GDP-dissociation inhibitor binding (GO:0051021)	3	2	.01	+	> 100	3.65E-05	3.86E-03
cyclin binding (GO:0030332)	34	3	.07	+	45.42	5.01E-05	5.19E-03
metal ion binding (GO:0046872)	4352	20	8.45	+	2.37	5.13E-05	5.20E-03
RNA polymerase II-specific DNA-binding transcription factor binding (GO:0061629)	349	6	.68	+	8.85	5.83E-05	5.80E-03
metalloendopeptidase activity (GO:0004222)	110	4	.21	+	18.72	6.93E-05	6.76E-03
cation binding (GO:0043169)	4443	20	8.63	+	2.32	6.98E-05	6.68E-03
protein serine kinase activity (GO:0106310)	362	6	.70	+	8.53	7.11E-05	6.68E-03
histone decrotonylase activity (GO:0160009)	5	2	.01	+	> 100	7.65E-05	7.06E-03
protein decrotonylase activity (GO:0160008)	5	2	.01	+	> 100	7.65E-05	6.93E-03
protein-containing complex binding (GO:0044877)	1762	12	3.42	+	3.51	8.98E-05	8.00E-03
SH2 domain binding (GO:0042169)	42	3	.08	+	36.77	9.05E-05	7.92E-03
tau protein binding (GO:0048156)	43	3	.08	+	35.92	9.67E-05	8.32E-03
non-membrane spanning protein tyrosine kinase activity (GO:0004715)	45	3	.09	+	34.32	1.10E-04	9.29E-03
sphingosine-1-phosphate receptor activity (GO:0038036)	8	2	.02	+	> 100	1.63E-04	1.36E-02
transcription factor binding (GO:0008134)	613	7	1.19	+	5.88	1.68E-04	1.38E-02
protein binding (GO:0005515)	14435	38	28.04	+	1.36	1.81E-04	1.46E-02
protein domain specific binding (GO:0019904)	661	7	1.28	+	5.45	2.66E-04	2.11E-02
histone H3 kinase activity (GO:0140996)	12	2	.02	+	85.80	3.29E-04	2.56E-02
DNA-binding transcription factor binding (GO:0140297)	490	6	.95	+	6.30	3.59E-04	2.76E-02
binding (GO:0005488)	16632	40	32.31	+	1.24	3.72E-04	2.81E-02
serine-type endopeptidase activity (GO:0004252)	180	4	.35	+	11.44	4.35E-04	3.25E-02
metallopeptidase activity (GO:0008237)	189	4	.37	+	10.90	5.21E-04	3.83E-02
phosphatase binding (GO:0019902)	191	4	.37	+	10.78	5.42E-04	3.93E-02
bioactive lipid receptor activity (GO:0045125)	16	2	.03	+	64.35	5.50E-04	3.93E-02

carbonate dehydratase activity (GO:0004089)	16	2	.03	+	64.35	5.50E-04	3.87E-02
transcription corepressor activity (GO:0003714)	196	4	.38	+	10.51	5.96E-04	4.14E-02
serine-type peptidase activity (GO:0008236)	198	4	.38	+	10.40	6.19E-04	4.24E-02
serine hydrolase activity (GO:0017171)	202	4	.39	+	10.19	6.66E-04	4.50E-02
identical protein binding (GO:0042802)	2189	12	4.25	+	2.82	6.77E-04	4.52E-02

TableS7. Analysis Type:PANTHER Overrepresentation Test (Released 20231017); Annotation Version and Release Date:GO Ontology database DOI: 10.5281/zenodo.8436609; Released 2023-10-09; Analyzed List: upload_1 (Homo sapiens); Reference List: Homo sapiens (all genes in database); Test Type: FISHER; Correction: FDR
