# Supplementary material for: HDAC6 as a Prognostic Factor and Druggable Target in HER2-Positive Breast Cancer
Source: Cancers (Basel). 2024 Nov 6;16(22):3752. doi: 10.3390/cancers16223752 (PMC11591923; doi:10.3390/cancers16223752)
Supplement: Supplementary file 1 [file cancers-16-03752-s001.zip › cancers-3285752-supplementary/TABLES/SupplementaryTableS8.pdf]

| Pathway identifier | Pathway name | #Entities found | #Entities total | Entities ratio | Entities pValue | Entities FDR | #Reactions found | #Reactions total | Reactions ratio | Species identifier | Species name | Submitted entities found |
|---|---|---|---|---|---|---|---|---|---|---|---|---|
| **R-HSA-9006934** | Signaling by Receptor Tyrosine Kinases | 19 | 631 | 0.04080444904293844 | 2,72E+03 | 1,93E+06 | 200 | 749 | 0.05087278407933166 | 9606 | Homo sapiens | ALK;PDGFRB;HDAC2;HSP90AA1;FLT1;HDAC3;DUSP3;ROCK2;SRC;SPHK1;PSEN2;BRAF;NCOR1;CHEK1;RAC1;JAK2;MET |
| **R-HSA-5663202** | Diseases of signal transduction by growth factor receptors and second messengers | 17 | 540 | 0.034919813760993276 | 3,27E+03 | 1,16E+07 | 136 | 512 | 0.0347755212932147 | 9606 | Homo sapiens | PDGFRB;ALK;HDAC4;HDAC2;HSP90AA1;HDAC3;SRC;PSEN2;BRAF;HDAC9;HDAC6;NCOR1;MDM2;RAC1;JAK2;MET |
| **R-HSA-350054** | Notch-HLH transcription pathway | 6 | 28 | 0.0018106570098292809 | 9,47E+04 | 2,23E+09 | 1 | 2 | 1,36E+12 | 9606 | Homo sapiens | HDAC4;HDAC2;NCOR1;HDAC3;HDAC9;HDAC6 |
| **R-HSA-162582** | Signal Transduction | 31 | 3036 | 0.19632695292291774 | 1,93E+06 | 3,42E+08 | 396 | 2539 | 0.1724512667255315 | 9606 | Homo sapiens | HDAC4;ALK;RET;HDAC2;HDAC3;FLT1;ROCK2;SRC;PSEN2;HDAC9;HDAC6;CHEK1;RAC1;JAK2;PDGFRB;HSP90AA1;MMP7;DUSP3;SYK;SPHK1;PLK1;MMP3;BRAF;NCOR1;CDK4;CDK2;MDM2;MET |

| | | | | | | | | | | | |
|---|---|---|---|---|---|---|---|---|---|---|---|
| **R-HSA-2644602** | Signaling by NOTCH1 PEST Domain Mutants in Cancer | 7 | 68 | 0.004397309881013968 | 5,36E+06 | 4,18E+09 | 2 | 21 | 0.001426339740542009 | 9606 | Homo sapiens | HDAC4;HDAC2;NCOR1;HDAC3;PSEN2;HDAC9;HDAC6 |
| **R-HSA-2644606** | Constitutive Signaling by NOTCH1 PEST Domain Mutants | 7 | 68 | 0.004397309881013968 | 5,36E+06 | 4,18E+09 | 2 | 21 | 0.001426339740542009 | 9606 | Homo sapiens | HDAC4;HDAC2;NCOR1;HDAC3;PSEN2;HDAC9;HDAC6 |
| **R-HSA-2894858** | Signaling by NOTCH1 HD+PEST Domain Mutants in Cancer | 7 | 68 | 0.004397309881013968 | 5,36E+06 | 4,18E+09 | 2 | 21 | 0.001426339740542009 | 9606 | Homo sapiens | HDAC4;HDAC2;NCOR1;HDAC3;PSEN2;HDAC9;HDAC6 |
| **R-HSA-2894862** | Constitutive Signaling by NOTCH1 HD+PEST Domain Mutants | 7 | 68 | 0.004397309881013968 | 5,36E+06 | 4,18E+09 | 2 | 21 | 0.001426339740542009 | 9606 | Homo sapiens | HDAC4;HDAC2;NCOR1;HDAC3;PSEN2;HDAC9;HDAC6 |
| **R-HSA-2644603** | Signaling by NOTCH1 in Cancer | 7 | 68 | 0.004397309881013968 | 5,36E+06 | 4,18E+09 | 2 | 39 | 0.0026489166610065883 | 9606 | Homo sapiens | HDAC4;HDAC2;NCOR1;HDAC3;PSEN2;HDAC9;HDAC6 |

| | | | | | | | | | | | | |
|---|---|---|---|---|---|---|---|---|---|---|---|---|
| **R-HSA-1980143** | Signaling by NOTCH1 | 7 | 84 | 0.0054319710294878843 | 2,25E+08 | 1,58E+09 | 4 | 39 | 0.0026489166610065883 | 9606 | Homo sapiens | HDAC4;HDAC2;NCOR1;HDAC3;PSEN2;HDAC9;HDAC6 |
| **R-HSA-2122947** | NOTCH1 Intracellular Domain Regulates Transcription | 6 | 57 | 0.0036859803414381 79 | 6,23E+07 | 3,99E+09 | 1 | 18 | 0.0012225769204645793 | 9606 | Homo sapiens | HDAC4;HDAC2;NCOR1;HDAC3;HDAC9;HDAC6 |
| **R-HSA-201556** | Signaling by ALK | 5 | 46 | 0.00297465080186239 | 7,16E+08 | 4,22E+11 | 28 | 42 | 0.002852679481084018 | 9606 | Homo sapiens | ALK;HDAC2;HDAC3;SRC |
| **R-HSA-156711** | Polo-like kinase mediated events | 4 | 23 | 0.001487325400931195 | 1,57E+10 | 8,48E+10 | 9 | 15 | 0.0010188141003871494 | 9606 | Homo sapiens | PLK1;CDC25A |
| **R-HSA-1643685** | Disease | 24 | 2637 | 0.1705250905328505 | 3,68E+09 | 1,84E+12 | 186 | 1927 | 0.1308836514297358 | 9606 | Homo sapiens | PDGFRB;ALK;HDAC4;HDAC2;HSP90AA1;HDAC3;SYK;ROCK2;SRC;PSEN2;BRAF;CYP19A1;HDAC9;CDC25A;HDAC6;NCOR1;CYP24A1;CDK4;CDK2;MDM2;RAC1;JAK2;MET |
| **R-HSA-** | Generic Transcription Pathway | 18 | 1586 | 0.1025607 | 5,30E | 2,49E | 73 | 884 | 0.06004211098 | 9606 | Homo sapiens | RET;HDAC4;HDAC2;HDAC3;SRC;HDAC9;HDAC6;NCOR1;CDK4;CHEK1;CDK2;MDM2;MET |

| | | | | | | | | | | | | |
|---|---|---|---|---|---|---|---|---|---|---|---|---|
| **212436** | | | | 8634247284 | +09 | +11 | | | 2816005 | | | |
| **R-HSA-6785807** | Interleukin-4 and Interleukin-13 signaling | 7 | 211 | 0.0136445938954992 23 | 1,01E+11 | 4,43E+10 | 19 | 47 | 0.00319228418121 3068 | 9606 | Homo sapiens | HSP90AA1;MMP3;PTGS2;JAK2 |
| **R-HSA-194138** | Signaling by VEGF | 6 | 140 | 0.0090532850491464 05 | 1,09E+11 | 4,47E+11 | 42 | 86 | 0.00584120084221 9656 | 9606 | Homo sapiens | HSP90AA1;FLT1;ROCK2;SRC;SPHK1;RAC1 |
| **R-HSA-9768919** | NPAS4 regulates expression of target genes | 4 | 41 | 0.0026513191929643 043 | 1,51E+11 | 5,90E+11 | 4 | 36 | 0.00244515384092 91586 | 9606 | Homo sapiens | RET;MDM2 |
| **R-HSA-73857** | RNA Polymerase II Transcription | 18 | 1728 | 0.1117434040351784 8 | 1,74E+11 | 6,01E+11 | 73 | 945 | 0.06418528832439 041 | 9606 | Homo sapiens | RET;HDAC4;HDAC2;HDAC3;SRC;HDAC9;HDAC6;NCOR1;CDK4;CHEK1;CDK2;MDM2;MET |
| **R-HSA-1257604** | PIP3 activates AKT signaling | 8 | 322 | 0.0208225556130 36 | 1,80E+11 | 6,01E+11 | 7 | 88 | 0.00597704272227 1276 | 9606 | Homo sapiens | PDGFRB;HDAC2;HDAC3;SRC;MDM2;RAC1;MET |

| | | | | | | | | | | | | |
|---|---|---|---|---|---|---|---|---|---|---|---|---|
| | | | | 732 | | | | | | | | |
| **R-HSA-8941326** | RUNX2 regulates bone development | 4 | 43 | 0.0027806518365235387 | 1,82E+11 | 6,01E+11 | 6 | 32 | 0.0021734700808259187 | 9606 | Homo sapiens | HDAC4;HDAC3;SRC;HDAC6 |
| **R-HSA-74160** | Gene expression (Transcription) | 19 | 1917 | 0.12396533885152612 | 1,89E+11 | 6,04E+11 | 80 | 1090 | 0.07403382462813285 | 9606 | Homo sapiens | RET;HDAC4;HDAC2;HSP90AA1;HDAC3;SRC;HDAC9;HDAC6;NCOR1;CDK4;CHEK1;CDK2;MDM2;MET |
| **R-HSA-8986944** | Transcriptional Regulation by MECP2 | 5 | 100 | 0.006466632177961717 | 3,01E+11 | 8,75E+11 | 8 | 77 | 0.005229912381987367 | 9606 | Homo sapiens | HDAC2;NCOR1;HDAC3;MET |
| **R-HSA-2682334** | EPH-Ephrin signaling | 5 | 102 | 0.006595964821520952 | 3,30E+10 | 8,75E+11 | 52 | 56 | 0.0038035726414453576 | 9606 | Homo sapiens | ROCK2;SRC;PSEN2;RAC1;EPHB4 |

Table S8. 25 most relevant pathways sorted by p-value.
