# Supplementary figures and images for: HDAC6 as a Prognostic Factor and Druggable Target in HER2-Positive Breast Cancer
Source: Cancers (Basel). 2024 Nov 6;16(22):3752. doi: 10.3390/cancers16223752 (PMC11591923; doi:10.3390/cancers16223752)

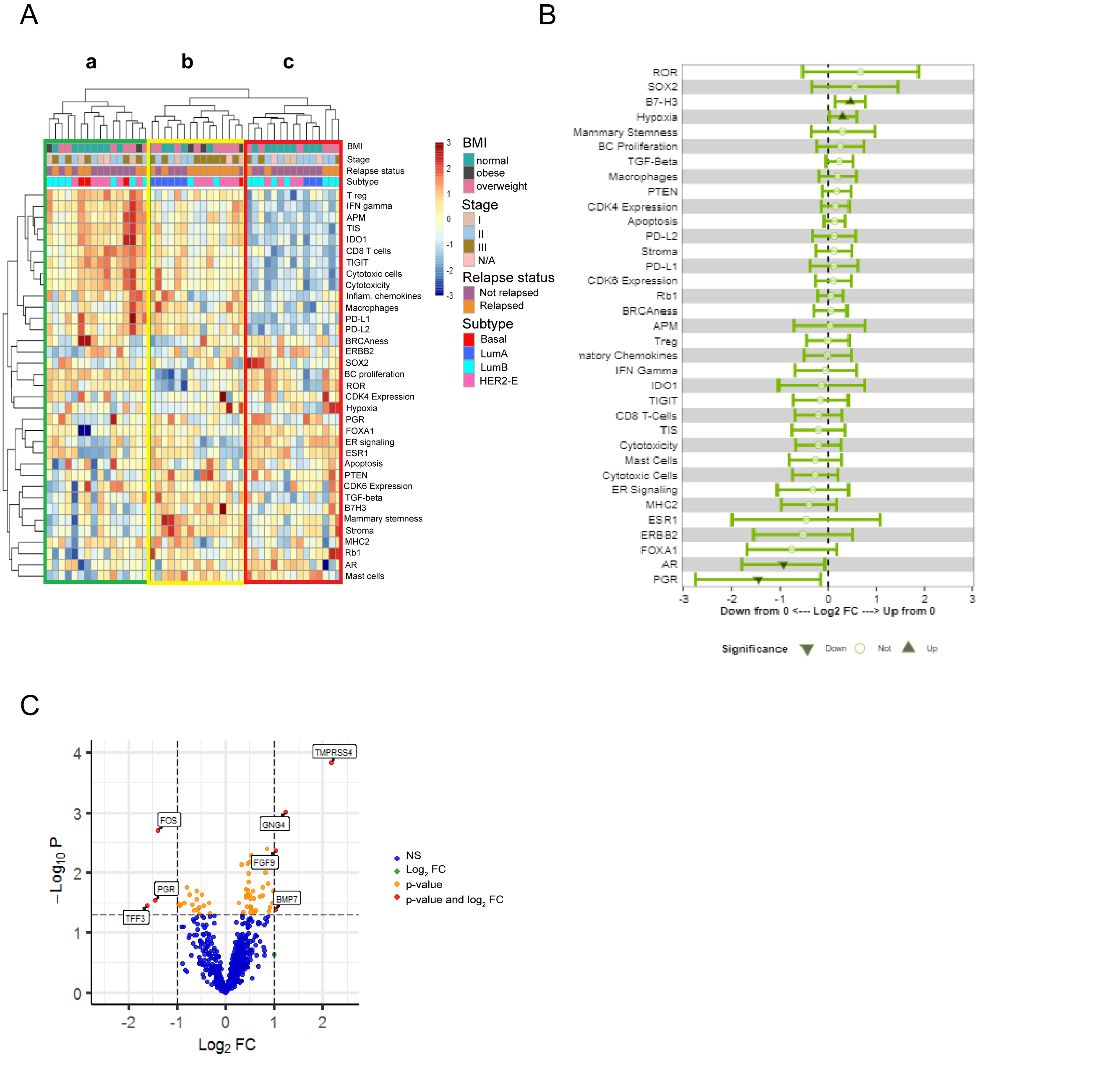

Supplement: Supplementary file 1 [file cancers-16-03752-s001.zip › cancers-3285752-supplementary/SupplementaryFigureS1.tif]

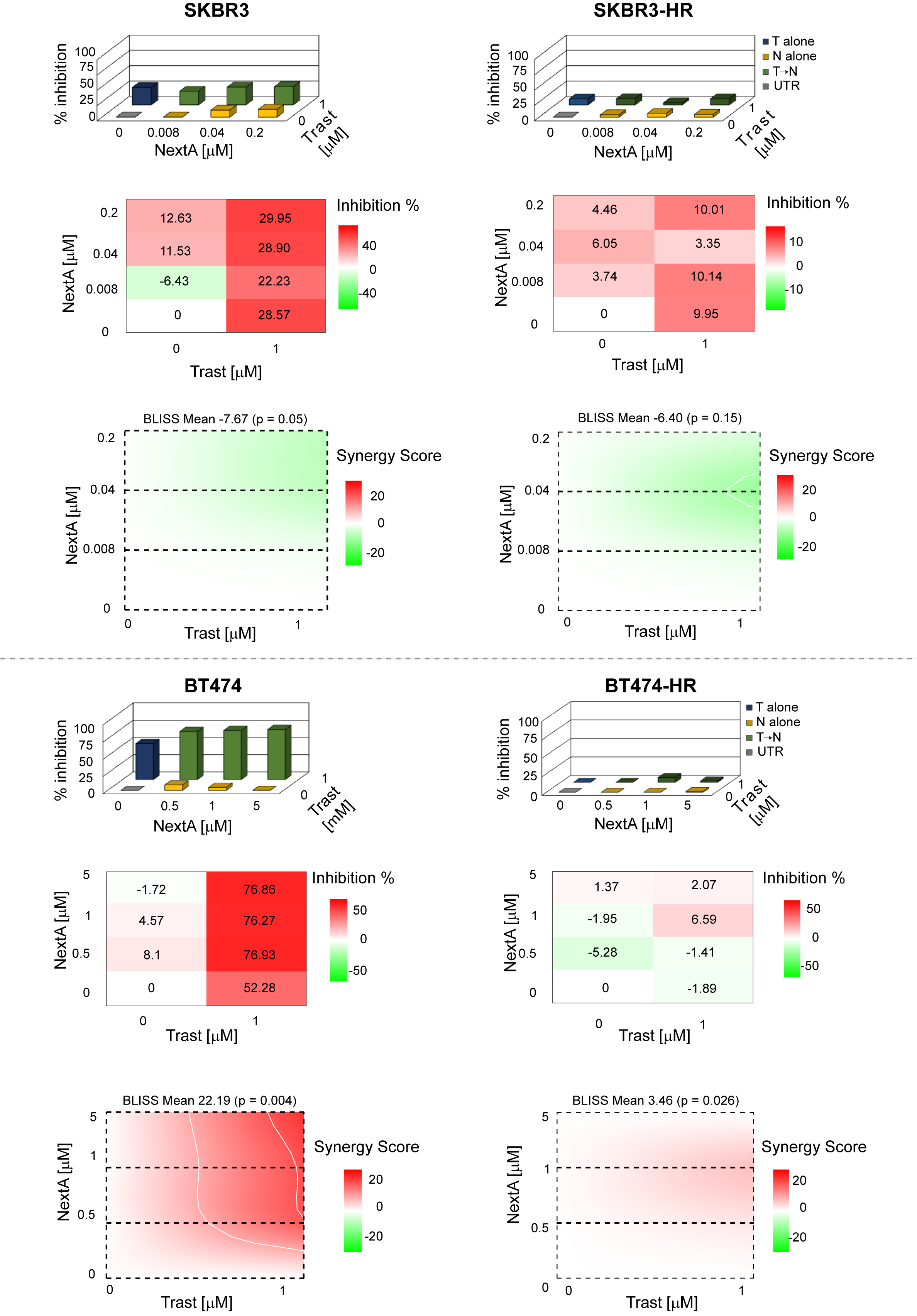

Supplement: Supplementary file 1 [file cancers-16-03752-s001.zip › cancers-3285752-supplementary/SupplementaryFigureS2.tif]

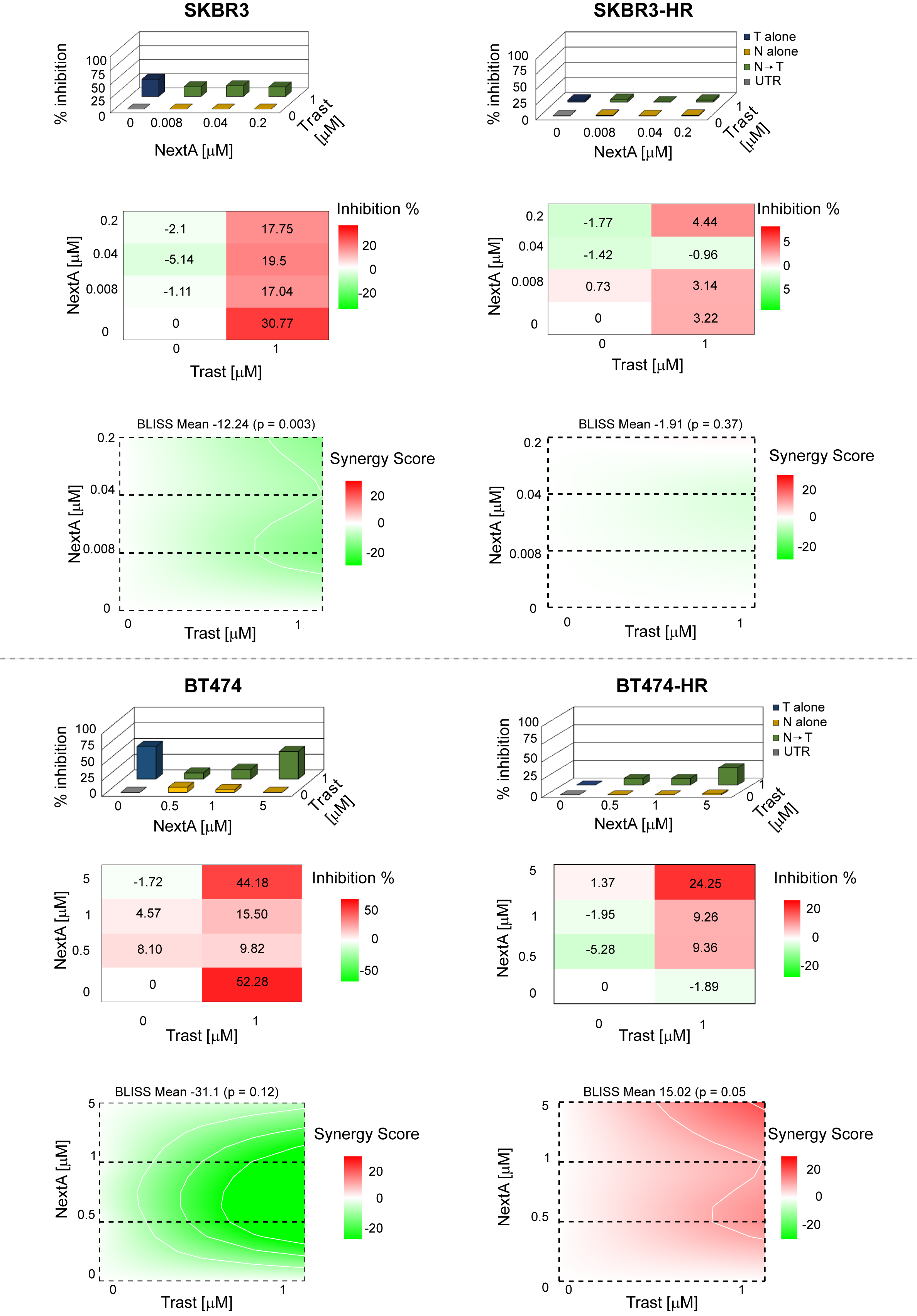

Supplement: Supplementary file 1 [file cancers-16-03752-s001.zip › cancers-3285752-supplementary/SupplementaryFigureS3.tif]
